# Impact of the transverse direction on the many-body tunneling dynamics in a two-dimensional bosonic Josephson junction

**DOI:** 10.1038/s41598-020-78173-w

**Published:** 2020-12-08

**Authors:** Anal Bhowmik, Sudip Kumar Haldar, Ofir E. Alon

**Affiliations:** 1 Department of Mathematics, University of Haifa, Haifa, 3498838 Israel; 2 Haifa Research Center for Theoretical Physics and Astrophysics, University of Haifa, Haifa, 3498838 Israel; 3 Department of Physics, SRM University Delhi-NCR, Plot No. 39 Rajiv Gandhi education city, Sonipat, 131029 India

**Keywords:** Physics, Ultracold gases

## Abstract

Tunneling in a many-body system appears as one of the novel implications of quantum physics, in which particles move in space under an otherwise classically-forbidden potential barrier. Here, we theoretically describe the quantum dynamics of the tunneling phenomenon of a few intricate bosonic clouds in a closed system of a two-dimensional symmetric double-well potential. We examine how the inclusion of the transverse direction, orthogonal to the junction of the double-well, can intervene in the tunneling dynamics of bosonic clouds. We use a well-known many-body numerical method, called the multiconfigurational time-dependent Hartree for bosons (MCTDHB) method. MCTDHB allows one to obtain accurately the time-dependent many-particle wavefunction of the bosons which in principle entails all the information of interest about the system under investigation. We analyze the tunneling dynamics by preparing the initial state of the bosonic clouds in the left well of the double-well either as the ground, longitudinally or transversely excited, or a vortex state. We unravel the detailed mechanism of the tunneling process by analyzing the evolution in time of the survival probability, depletion and fragmentation, and the many-particle position, momentum, and angular-momentum expectation values and their variances. As a general rule, all objects lose coherence while tunneling through the barrier and the states which include transverse excitations do so faster. In particular for the later states, we show that even when the transverse direction is seemingly frozen, prominent many-body dynamics in a two-dimensional bosonic Josephson junction occurs. Implications are briefly discussed.

## Introduction

After the experimental observations of Bose–Einstein condensation (BEC)^1–3^, ultra-cold quantum gases have emerged as one of the most advanced platforms to mimic a wide variety of typical models of condensed-matter physics, optics, high-energy physics, and even of quantum biology and chemistry^4–11^. One of such well known paradigms of quantum physics is the existence of Josephson effect which is a clear manifestation of the macroscopic quantum coherence, originally predicted for superconductors^12^ and later observed in superfluid $^3$He^13^ and gaseous BECs^14,15^. When ultra-cold bosons are tunneling in a double-well potential, the system is usually referred to as bosonic Josephson junction (BJJ)^16^.

An extensive theoretical study of trapped BECs in one-dimensional double-well potentials is available using a variety of theoretical approaches^17–30^. Some of the interesting features, such as Josephson oscillations^14,28,31,32^ and self trapping^14,32^ have been reported using a two-mode theory. Also, one-dimensional Josephson oscillations are studied using a quantum-Langevin approach and the exact Tonks–Girardeau solution in the impenetrable-boson limit^33^. The quantum tunneling of two one-dimensional quasi-condensates have been investigated recently theoretically^34^ as well as experimentally^35^. A full many-body Schrödinger dynamics starting from the ground state of the BEC in one of the wells (hereafter for brevity, ground state of the BEC) has been studied in one-dimensional double-well potentials and shows the development of fragmentation and loss of coherence in the BEC^36–40^. The uncertainty product of the many-particle position and momentum operators^41^ as well as the evolution in time of the position and momentum variances^40^ have been studied by solving the full many-body Schrödinger equation. The development of fragmentation of the ground state of the BEC validates the necessity of a many-body treatment in order to obtain the accurate dynamical behavior of bosons in the BJJ.

Tunneling dynamics of the ground state of a trapped BEC in higher dimensions has also been explored using two-mode or improved two-mode models^42,43^. Ananikian and Bergeman showed that when the extent of the wave function in each well vary appreciably with time, the nonlinear interaction term creates a temporal change in the tunneling energy or rate^42^. Spagnolli et al. reported a detailed study of the transition from Rabi to plasma oscillations by crossing over from the attractive to repulsive inter-atomic interaction in terms of the evolution of atomic imbalance^43^. Moreover, in two dimensions (2D), the tunneling dynamics of trapped vortices were studied using Gross–Pitaevskii mean-field model in 2D superfluids^44^, in an harmonic potential with a Gaussian potential barrier^45^, and between two pinning potentials^46^. Salgueiro et al. proposed a method of generating replicas of a vortex state in a double-well potential formed by conjoining two Gaussian potentials using the mean-field approach^47^. Garcia-March and Carr showed a comparative study of the tunneling of axisymmetric and transverse vortex structures^48^. The most of the literature in relation with ground and vortex states of a BEC in a two-dimensional double-well potential are devoted to the density oscillations between the wells. There is a recent study of tunneling dynamics of the vortex state using an in-principle numerically-exact many-body theory in a 2D radial double-well trap^49^. Beinke et al. showed that the development of the fragmentation of the vortex state is accompanied by damping of the amplitude of the survival probability, thereby indicating the importance of the accurate many-body theoretical treatment^49^. Moreover, on a different note, the hidden vortices in a rotating double-well potential^50^, excitation of non-zero angular-momentum modes in tunnel-coupled two-dimensional Bose gas^51^, the creation of vortices in a BEC by external laser beam with orbital angular-momentum^52–54^, and the dissipative dynamics in an atomic Josephson junction between Fermi superfluids^55,56^ are studied in the literature. Although there is some literature discussing the dynamics of the ground and vortex states in a double-well potential, there is no detailed investigation of the inter-connection of the density oscillations with the time evolution of quantum mechanical observables and their variances, let alone beyond one spatial dimension. Furthermore, to the best of our knowledge, there is no available literature which discusses the tunneling dynamics of complicated bosonic objects in a double-well potential by solving the many-particle problem at the many-body level of theory.

The main focus of this work is to explain the physics behind the tunneling dynamics of a few intricate bosonic clouds in a 2D double-well potential by analyzing the time evolution of various physical quantities, focusing on tunneling scenarios and research questions which require at least a 2D geometry to investigate. In order to explore the tunneling dynamics in a 2D symmetric double-well with the junction along the *x* direction, we consider four basic structures of bosonic clouds in the harmonic potential, namely, ground, *x*-excited, *y*-excited, and vortex states. Per definition, the *y*-excited and vortex states have no one-dimensional analogs. Although, there are one-dimensional analogs to the ground and *x*-excited states, we ask how the inclusion of the transverse direction can affect the overall dynamics of all four initial states. A general question we ask is if there is any difference between the many-body and mean-field dynamics in the 2D BJJ. We ask whether and how quantum correlations develop in the process of tunneling for the initial states considered here. Will there be any qualitative and quantitative differences in the correlations due to the different initial structures of the bosonic clouds? Therefore, to investigate the tunneling dynamics in detail in a 2D double-well, we need to solve the many-body Schrödinger equation numerically accurately. A particularly suitable approach to solve the full Schrödinger equation is called the multiconfigurational time-dependent Hartree for bosons (MCTDHB) method^57–59^.

In this paper, we show that the ground and excited states can tunnel through the barrier without destroying their initial structures. But the vortex state creates two vortex dipoles in the tunneling process and the dipoles rotate around the minima of the respective well. We find that the creation of the dipoles from the vortex states relies on the tunneling of the excited states considered here. We observe a difference between the mean-field and many-body density oscillations in the long-time dynamics for all objects due to the growing degree of quantum correlations in the later. We show that the fragmentation develops faster when there is transverse excitation in the system. Moreover, the mechanism of the development of fragmentation exhibits significant differences when there are transverse excitations. All in all, we have studied the time evolution of a purely many-body quantity, fragmentation, and discussed its impact on the survival probability, expectation values and variances. We find an interconnection between the density oscillations and some quantum mechanical quantities by accurately calculating the time evolution of the survival probability and the many-particle position, momentum, and angular-momentum expectation values and their variances, both at the mean-field and many-body levels. As the variance is a sensitive probe of correlations^60^, even when the bosons are fully condensed, comparisons of the mean-field and many-body variances show that the correlations have different impact on the different physical quantities depending on the initial structure of the bosonic cloud and the presence of transverse excitations in the system. Moreover, as the ground and longitudinally-excited states have one-dimensional manifestations, to show the impact of the transverse direction we compare the dynamics of different quantum mechanical quantities found in two-dimensional double-well potential and their one-dimensional analogs.

## System and methodology

According to the time-dependent many-body Schrödinger equation, the dynamics of *N* interacting structureless bosons are governed by

1 $$\begin{aligned} {\hat{H}}\Psi = i\dfrac{\partial \Psi }{\partial t}, \qquad {\hat{H}}(\mathbf{r }_1, \mathbf{r }_2,\ldots , \mathbf{r }_N)= \sum _{j=1}^{N} {\hat{h}}(\mathbf{r }_j)+\sum _{j<k} {\hat{W}}(\mathbf{r }_j-\mathbf{r }_k). \end{aligned}$$

Here, ${\hat{h}}(\mathbf{r })={\hat{T}}(\mathbf{r })+{\hat{V}}(\mathbf{r })$ is the one-particle Hamiltonian where ${\hat{T}}(\mathbf{r })$ and ${\hat{V}}(\mathbf{r })$ represent the kinetic energy and trap potential, respectively. ${\hat{W}}(\mathbf{r }_j-\mathbf{r }_k)$ is a short-range repulsive inter-particle interaction modeled by a Gaussian function, $W(\mathbf{r }_1-\mathbf{r }_2)=\lambda _0\dfrac{e^{-(\mathbf{r }_1-\mathbf{r }_2)^2/2\sigma ^2}}{2\pi \sigma ^2}$ with $\sigma =0.25\sqrt{\pi }$, to avoid the regularization problems of the zero-ranged contact potential in two spatial dimensions^49,61–64^, also see supplemental material for the one-dimensional analog of the interaction. The particular shape of the inter-particle interaction model potential does not impact the physics of the bosons to be described below. To quantify the interaction strength, the mean-field interaction parameter $\Lambda =\lambda _0(N-1)$ is standardly introduced. Throughout this work, $\mathbf{r }=(x,y)$ is the position vector in two spatial dimensions and the natural units $\hbar =m=1$ are employed.

We solve the time-dependent many-boson Schrödinger equation presented in Eq. (1) using the MCTDHB method^36,39,40,49,57,58,61,65–73,73–82^. The method is well documented and applied in the literature^59^. Detailed derivation of the MCTDHB equations of motion is described in^58^. For our numerical computations, we use the numerical implementation in^83,84^. MCTDHB uses the ansatz

2 $$\begin{aligned} |\Psi (t)\rangle =\sum _{{\{n\}}}C_\mathbf{n }(t)|\mathbf{n };t\rangle \end{aligned}$$

where $|\mathbf{n };t\rangle = |n_1,n_2,\ldots ,n_M;t\rangle$ are the time-dependent permanents obtained by distributing *N* bosons in *M* time-adaptive single-particle orbitals. In the limit $M\rightarrow \infty$, the permanents $|\mathbf{n };t\rangle$ span the complete $N-$particle Hilbert space and the expansion in Eq. (2) becomes formally exact. The usage of time-adaptive permanents allows one to solve the time-dependent Schrödinger equation numerically accurately with finite, often quite small number of orbitals *M*^85^. At the opposite end, for $M=1$, Eq. (2) becomes the Gross–Pitaevskii ansatz and solves the time-dependent Gross–Pitaevskii equation. In other words, for $M=1$ Eq. (2) boils down to the time dependent Gross–Pitaeveskii equation, $\Psi _{\text {GP}}(\mathbf{r }_1, \ldots ,\mathbf{r }_N;t)=\prod _{j=1}^{N}\phi _j(\mathbf{r },t)$, and accordingly the MCTDHB equations of motion^58^ reduce to $[{\hat{h}}(\mathbf{r })+(N-1) \int d\mathbf{r }^\prime |\phi (\mathbf{r }^\prime , t)|^2{\hat{W}}(\mathbf{r }-\mathbf{r }^\prime )]\phi (\mathbf{r }, t)=i\dfrac{\partial \phi (\mathbf{r }, t)}{\partial t}$^59^.

The main theme of this work is to explore the dynamical behavior of the ground, longitudinally and transversely excited, and vortex states in a symmetric 2D double-well in terms of different physical quantities such as the survival probability, depletion and fragmentation, and the many-particle position, momentum, and angular-momentum variances. These would help us to extract relevant information embedded in the *N*-boson time-dependent wavefunction and shed light on the physics of tunneling in the junction. We begin our analysis by preparing the initial state either as the ground ($\Psi _G$), *x*-excited ($\Psi _X$) or *y*-excited ($\Psi _Y$), or a linear combination of $\Psi _X$ and $\Psi _Y$, i.e. a vortex state ($\Psi _V$), of non-interacting many bosons at the left well of a 2D symmetric double-well potential. The double well potential is formed by fusing together two harmonic potentials, $V_L(x,y)=\dfrac{1}{2}(x+2)^2+\dfrac{1}{2}y^2$ and $V_R(x,y)=\dfrac{1}{2}(x-2)^2+\dfrac{1}{2}y^2$, where $V_L(x,y)$ and $V_R(x,y)$ represent the left and right wells of the trap potential, respectively, with a quadratic polynomial $\dfrac{3}{2}(1-x^2)+\dfrac{1}{2}y^2$ in the region $|x|\le \dfrac{1}{2}$ and $-\infty< y<\infty$, and is given by

3 $$\begin{aligned} V_T(x,y)= {\left\{ \begin{array}{ll} \dfrac{1}{2}(x+2)^2+\dfrac{1}{2}y^2, x<-\dfrac{1}{2}, -\infty< y<\infty , \\ \dfrac{3}{2}(1-x^2)+\dfrac{1}{2}y^2, |x|\le \dfrac{1}{2}, -\infty< y<\infty , \\ \dfrac{1}{2}(x-2)^2+\dfrac{1}{2}y^2, x>+\dfrac{1}{2}, -\infty< y<\infty . \end{array}\right. } \end{aligned}$$

**Figure 1:**
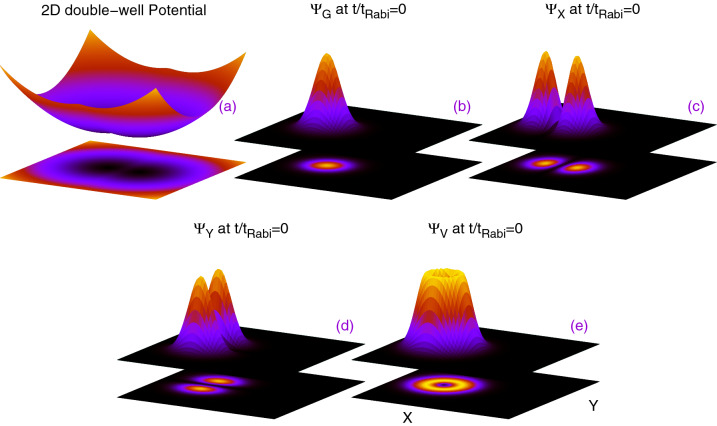
Schematic diagrams for (**a**) the 2D double-well potential described in Eq. (3) and the initial density distributions for (**b**) $\Psi _G$, (**c**) $\Psi _X$, (**d**) $\Psi _Y$, and (**e**) $\Psi _V$. The *x*- and *y*-axes are shown in panel (**e**). The quantities shown are dimensionless.

$V_T(x,y)$ (see Fig. 1a) is a natural 2D generalization of the one-dimensional potential used, e.g., in^41^. The mathematical forms of the initial conditions, $\Psi _G$, $\Psi _X$, $\Psi _Y$, and $\Psi _V$, are taken as $\Psi _G=\dfrac{1}{\sqrt{\pi }}F(x,y)$, $\Psi _X=\sqrt{\dfrac{2}{\pi }}(x+2)F(x,y)$, $\Psi _Y=\sqrt{\dfrac{2}{\pi }}y F(x,y)$, and $\Psi _V=\frac{1}{\sqrt{2}}(\Psi _X+i\Psi _Y)$, where $F(x,y)=exp[-\{(x+2)^2+y^2\}/2]$, and their initial density distributions are shown in Fig. 1. Here, $\Psi _G$ and $\Psi _X$ can have one-dimensional analogs, therefore, to study the different quantum mechanical properties using these states in their one-dimensional analogs, one has to decouple the transverse direction from the longitudinal direction. The derivation to move from two dimensions to one dimension for $\Psi _G$ and $\Psi _X$ is included in the supplemental material.

In order to investigate the time evolution of the prepared initial states, we suddenly quench the inter-particle interaction at $t=0$ from $\Lambda =0$ to $\Lambda =0.01\pi$ accompanied by the change of trapping potential from the initial single-well, $V_L(x,y)$, to the final double-well, $V_T(x,y)$, potential. The consistency of the initial-state preparation is discussed in the supplemental material. Now, we will investigate the tunneling dynamics of the considered bosonic clouds in the symmetric double-well potential $V_T(x,y)$.

## The tunneling dynamics and its analysis

In this section, we explore in detail the time evolution of various physical quantities for a collection of bosons trapped in a 2D symmetric double-well. In particular, we are interested to show the time variation of the survival probability in the left well, the degree of fragmentation of the bosons, and the expectation values and variances of the position, momentum, and angular-momentum many-particle operators for the initial states, $\Psi _G$, $\Psi _X$, $\Psi _Y$, and $\Psi _V$. These quantities draw increasingly more involved information from the time-dependent many-boson wave function, namely, from the density, reduced one-particle density matrix, and reduced two-particle density matrix, respectively. As the double-well potential is symmetric, preparation of the initial state either in left or right well does not affect the quantities discussed here.

Our research approach is a combined investigation of the dynamics at the mean-field and many-body levels of theory. The MCTDHB theory incorporates the correlations among the bosons, therefore to highlight the many-body effects, we compare the many-body survival probability, expectation values, and variances computed using the MCTDHB method with the corresponding mean-field ($M=1$ time-adaptive orbitals) results. In our work, we have performed all the many-body computations for $\Psi _{G}$ and $\Psi _{X}$ using $M=6$ time-adaptive orbitals, while for $\Psi _{Y}$ and $\Psi _{V}$ using $M=10$ time-adaptive orbitals. We shall see later on that the inclusion of the transverse excitations generally requires more time-adaptive orbitals to faithfully represent the many-body dynamics. In order to check the convergence with respect to the orbital numbers, we have repeated our computations with $M=10$ orbitals for $\Psi _{G}$ and $\Psi _{X}$ and with $M=12$ orbitals for $\Psi _{Y}$ and $\Psi _{V}$; all the results are found to be well converged, see the supplemental material for more details. For the numerical solution, we use a grid of $64^2$ points in a box of size $[-10, 10) \times [-10, 10)$ with periodic boundary conditions. Convergence of the results with respect to the number of grid points has been verified using a grid of $128^2$ points, see the supplemental material. All many-body computations are carried out for a finite number of bosons, $N=10$, with the inter-boson interaction $\Lambda =0.01\pi$. The mean-field computations are done for the same interaction parameter $\Lambda =0.01\pi$. Therefore, one can relate the tunneling dynamics of the bosonic clouds between the many-body and mean-field levels. Furthermore, it is instructive to mention that all the systems mentioned here are weakly interacting, which allows us to mimic the so-called infinite-particle limit of the interacting bosons, at least for very short times. We set the time-scale for the dynamics equal to the period of the Rabi oscillations $(t_{\text {Rabi}})$ in the double well trap presented in Eq. (3). Here $t_{\text {Rabi}}=\dfrac{2\pi }{\Delta E}=132.498$, where $\Delta E$ is the energy difference between the ground state and first excited state, calculated by diagonalizing the single-particle Hamiltonian using discrete variable representation method. The ground and excited states in the 2D double-well are the even and odd functions along the *x*-direction. We shall use the same time scale for all tunneling processes discussed below, to facilitate a direct comparison between them.

### Density and survival probability

**Figure 2:**
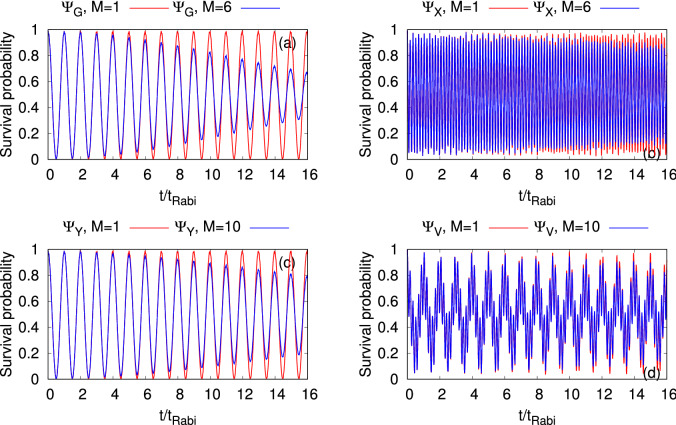
Time evolution of the survival probability in the left well, $P_L(t)$, of a symmetric 2D double-well potential for the initial states (**a**) $\Psi _G$, (**b**) $\Psi _X$, (**c**) $\Psi _Y$, and (**d**) $\Psi _V$. Mean-field results are in red solid line and corresponding many-body results are in blue solid line. The interaction parameter is $\Lambda =0.01\pi$ and the number of bosons is $N=10$. The many-body time evolutions are computed using the MCTDHB method with $M=6$ time-adaptive orbitals for $\Psi _{G}$ and $\Psi _{X}$ and $M=10$ time-adaptive orbitals for $\Psi _{Y}$ and $\Psi _{V}$. See the text for more details. The quantities shown are dimensionless.

**Figure 3:**
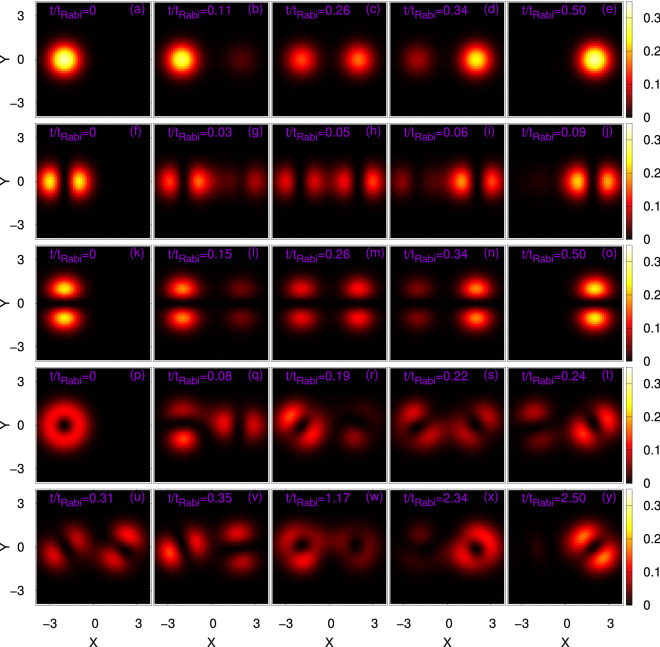
Time evolution of the many-body density oscillations in a symmetric 2D double-well. The interaction parameter is $\Lambda =0.01\pi$ and the number of bosons is $N=10$. Shown are the densities per particle for the initial states $\Psi _G$ (first row, from left to right, at $\frac{t}{t_{Rabi}}=0, 0.11, 0.26, 0.34$, and 0.50); $\Psi _X$ (second row, at $\frac{t}{t_{Rabi}}=0, 0.03, 0.05, 0.06$, and 0.09); $\Psi _Y$ (third row, at $\frac{t}{t_{Rabi}}=0, 0.15, 0.26, 0.34$, and 0.50); and $\Psi _V$ (fourth and fifth rows, at $\frac{t}{t_{Rabi}}=0, 0.08, 0.19, 0.22, 0.24$ and 0.31, 0.35, 1.17, 2.34, 2.50). The many-body time evolutions are computed using the MCTDHB method with $M=6$ time-adaptive orbitals for $\Psi _{G}$ and $\Psi _{X}$ and $M=10$ time-adaptive orbitals for $\Psi _{Y}$ and $\Psi _{V}$. See the text for more details. The quantities shown are dimensionless.

We begin our investigation with the time evolution of the most basic quantity, the density $\rho (x,y;t)$ of the bosonic clouds $\Psi _G$, $\Psi _X$, $\Psi _Y$, and $\Psi _V$. To this end, we present the interconnection between the four densities with respect to the survival probability in the left well, $P_L(t)=\int \limits _{x=-\infty }^{0}\int \limits _{y=-\infty }^{+\infty }dx dy \dfrac{\rho (x,y;t)}{N}$. In Fig. 2, we compare the many-body dynamics of $P_L(t)$ with the corresponding mean-field results. In order to have a more detailed description of the tunneling dynamics of the considered initial states, we depict surface plots of the density oscillations at the many-body level in Fig. 3. We observe in Fig. 2 the tunneling of the density back and forth between the left and right wells for all bosonic clouds, but the frequency of the tunneling oscillations are distinct for the different initial states, apart from $\Psi _G$ and $\Psi _Y$ which have essentially the same frequency of oscillations. The tunneling dynamics are consistent with the density oscillations shown in Fig. 3. For a particular initial state the frequency of the tunneling oscillations is practically identical at the mean-field and many-body levels, but certainly it does not remain so for the amplitudes of the tunneling oscillations in the course of time evolution. As $\Psi _G$ and $\Psi _Y$ both lie (in the non-interacting system) in the lowest band along the *x*-direction (the direction along which the barrier is formed), the tunneling oscillations of $\Psi _Y$ are very similar to those of $\Psi _G$ at the mean-field level, both in frequency and in amplitude. On the other hand, $\Psi _X$ lies (in the non-interacting system) in the first excited band along the *x*-direction, it ’feels’ a smaller potential barrier, and therefore its tunneling oscillations are faster. Furthermore, the effect of coupling between the lowest energy band and the higher excited states produces high-frequency breathing oscillations for $\Psi _X$.

We observe the complete tunneling of bosons from the left well to the right well without destroying the structure of the initial states in Fig. 3 for $\Psi _G$, $\Psi _X$, and $\Psi _Y$ at about $t=0.50 t_{\text {Rabi}}$, $t=0.09 t_{\text {Rabi}}$ and $t=0.50 t_{\text {Rabi}}$, respectively. Recall the interaction is weak $(\Lambda =0.01\pi )$ and the tunneling period is very close to that of non-interacting bosons. It is clear from Figs. 2 and 3 that the nature of tunneling for the vortex state is very different and intricate compared to the other initial bosonic clouds. As $\Psi _V$ is a linear combination of $\Psi _X$ and $\Psi _Y$ at $t=0$ and the interaction is weak, the tunneling of $\Psi _V$ can be interpreted by combining the resulting dynamics of $\Psi _X$ and $\Psi _Y$. In Fig. 3, it is shown that the vortex state initially destroys its structure in the process of tunneling and creates two dipole states. It is noted that the essentially full tunneling of $\Psi _V$ happens at about $t=2.50 t_{\text {Rabi}}$ but partial tunneling (around 95%) of $\Psi _V$ is observed at about $t=0.50 t_{\text {Rabi}}$. With progress of time, the dipole structures rotate and change their relative phase. The collapse of the vortex structure into dipoles and the rotation of these dipoles take place due to the different tunneling frequencies of $\Psi _X$ and $\Psi _Y$. At $t=2.50 t_{Rabi}, \Psi_V$ practically tunnels to the right well. It is one of that definite moments in time when each of the clouds of $\Psi _X$ and $\Psi _Y$ individually and practically completely tunnels to the right well after around 14 and 2.5 oscillations, respectively. Similarly, in the mean-field, $\Psi _X$, $\Psi _Y$ and hence $\Psi _V$ completely tunnel back to the left well at about $t=5.00 t_{Rabi}$ (not shown).

The beginning of the time evolution in all panels of Fig. 2 shows a complete overlap between the mean-field and many-body results, thereby confirming that the survival probability can be accurately described by the mean-field theory at short time scales for the interaction strength considered here. Correspondingly, the densities per particle computed at the many-body level in Fig. 3 match the mean-field densities per particles of the bosonic clouds. This situation emulates the so-called infinite-particle limit of the time-dependent many-boson Schrödinger equation, in which the time-dependent density per particle coincides with the respective density obtained from the time-dependent Gross-Pitaevskii equation, see in this respect^41,86^. As time progresses, we observe incomplete tunneling of the densities of all the systems at the many-body level, a first signature of the build up of many-body correlations, resulting in a gradual decrease in the amplitudes of the oscillations. The decay in the amplitudes of the tunneling oscillations can not be seen at the level of mean-field theory. The results generalize what is known in the literature for tunneling from the ground state (of the left well) in BJJs^36^. Looking at Fig. 2, the decay rates of the density oscillations for $\Psi _X$ and $\Psi _V$ are rather similar and quite smaller from the decay rates of $\Psi _G$ and $\Psi _Y$. The intuition suggests that since $\Psi _X$ and $\Psi _V$ ’feel’ a smaller barrier when tunneling, many-body effects would develop slower, and hence the above-discussed decay rates are smaller. Correspondingly, $\Psi _G$ has the highest decay rate and hence reaches the smallest amplitude of oscillations at the largest time presented here. As $\Psi _G$ and $\Psi _X$ have one-dimensional manifestations, we compare the survival probabilities of these states calculated in two-dimensional double-well potential with their respective one-dimensional analogs both at the mean-field and many-body levels, and we find that the results are essentially identical in terms of amplitude and frequency due to the treatment of weakly interacting system (see Fig. S11 of supplemental material).

We shall analyze further measures and signatures of the many-body dynamics, and impact of the transverse direction, and return to this intuitive reasoning in the next subsection. As the transversely-excited and vortex states cannot be created in one spatial dimensions, it will be particularly interesting to dig deeper into the many-body as well as the mean-field dynamics of the $\Psi _Y$ and $\Psi _V$ states of BECs in the two-dimensional geometry. Furthermore, we shall be looking for signatures originating from the impact of the transverse direction in the tunneling dynamics of $\Psi _G$ and $\Psi _X$ which do have one-dimensional analogs.

### Dynamics of the condensate fraction and fragmentation

We have already found a difference between the mean-field and many-body time developments of the survival probability, $P_L(t)$. This difference implies that there are many-body correlations which gradually appear in the tunneling process. To study the effect of the quantum correlations on the tunneling dynamics, we would like to discuss how the depletion or fragmentation emerges, depending on the shape of the different initial states. To this end, we compute the reduced one-particle density matrix from the time-dependent many-boson wave-function (Eq. 2) and diagonalize the former for obtaining the time-dependent occupation numbers $n_j(t)$ and natural orbitals $\phi _j(x,y;t)$^87,88^. Here we present the time evolution of the condensate fraction, $\dfrac{n_1(t)}{N}$, and the details of the depletion $\dfrac{n_{j>1}(t)}{N}$ of the initial states in terms of the occupation numbers of the natural orbitals. The change in the occupation number of the first natural orbital signifies the loss of coherence in the initial state. We use the term fragmentation in a broad manner, to indicate a large amount of depletion, rather than only in its strict meaning of a macroscopic occupation of more than a single natural orbital.

**Figure 4:**
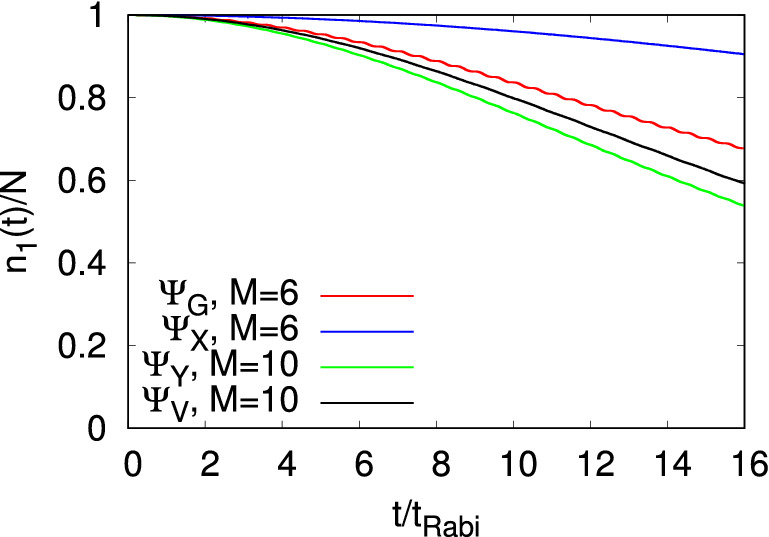
Time-dependent condensate fraction, $n_1(t)/N$, in a symmetric 2D double-well for the initial states $\Psi _G$, $\Psi _X$, $\Psi _Y$, and $\Psi _V$. The number of bosons is $N=10$ and the interaction parameter $\Lambda =0.01\pi$. The results have been obtained by the MCTDHB method with $M=6$ time-adaptive orbitals for $\Psi _{G}$ and $\Psi _{X}$ and $M=10$ time-adaptive orbitals for $\Psi _{Y}$ and $\Psi _{V}$. See the text for more details. Color codes are explained in the panel. The quantities shown are dimensionless.

**Figure 5:**
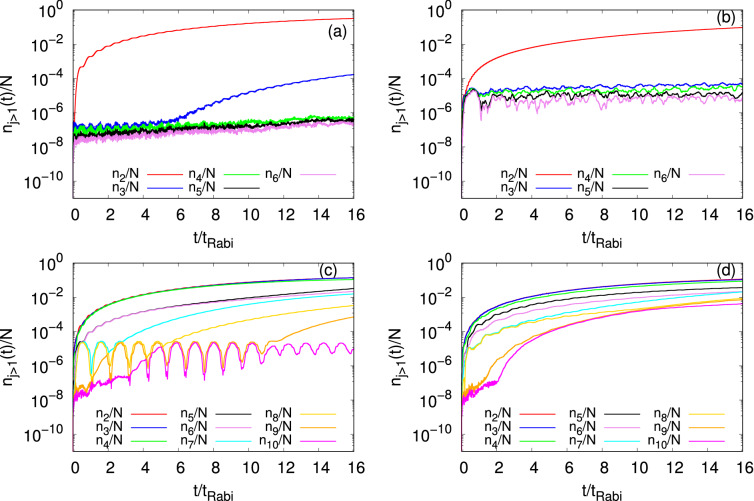
Details of the depletion. Time evolution of the occupation numbers per particle of the higher natural orbitals, $n_{j>1}(t)/N$, in a symmetric 2D double-well for the initial states (**a**) $\Psi _G$, (**b**) $\Psi _X$, (**c**) $\Psi _Y$, and (**d**) $\Psi _V$. The results have been obtained by the MCTDHB method with $M=6$ time-adaptive orbitals for $\Psi _{G}$ and $\Psi _{X}$ and $M=10$ time-adaptive orbitals for $\Psi _{Y}$ and $\Psi _{V}$. See the text for more details. Color codes are explained in the panel. The quantities shown are dimensionless.

Figure 4 presents the time-dependent occupation of the first natural orbital for the initial states $\Psi _G$, $\Psi _X$, $\Psi _Y$, and $\Psi _V$. The corresponding occupations of the higher natural orbitals are collected in Fig. 5. As the MCTDHB computations have been performed with $M=6$ self-consistent orbitals for $\Psi _{G}$ and $\Psi _{X}$ and $M=10$ self-consistent orbitals for $\Psi _{Y}$ and $\Psi _{V}$, we have plotted the occupancies of the higher natural orbitals to have a comparative study among all the initial states and show how coherence is lost. For the dynamics at longer times and convergence of the individual occupation numbers see the supplemental material. Overall, here it is observed that as time increases the occupation of the first natural orbital decreases with a weak oscillatory background, and the occupations of all the higher natural orbitals gradually increase, generally and in particular the lower ones in an oscillatory manner. Some of the smaller ones, e.g., for $\Psi _Y$, are oscillatory first and then increasing. The oscillatory background atop of the global time-evolution of the occupation numbers is in reminiscence of the tunneling back and forth in the junction. Furthermore, one can see some high-frequency oscillations in the profiles of the natural occupancy of the higher orbitals. These types of oscillations are the consequence of the time-dependent density oscillations. Figures 4 and 5 demonstrate that all the initial states start depleting and eventually become fragmented with time. Also, we find that $\dfrac{n_1(t)}{N}$ of $\Psi _G$ and $\Psi _X$ in two-dimensional double-well fall on top of the corresponding results of one-dimensional analogs (see Fig. S12 of supplemental material).

Examination of the respective occupation numbers of $\Psi _G$, $\Psi _X$, $\Psi _Y$, and $\Psi _V$ reveal a few trends. The first and perhaps the most prominent one, is that transverse excitation enhances fragmentation. Indeed, as time passes by, $\Psi _Y$ losses coherence faster than $\Psi _G$ and, analogously, $\Psi _V$ losses coherence faster than $\Psi _X$, see Fig. 4. On the other hand, longitudinal excitations suppress fragmentation, namely, $\Psi _X$ losses coherence slower than $\Psi _G$ and, similarly, $\Psi _V$ losses coherence slower than $\Psi _Y$. All in all, $\Psi _Y$ is the fastest to fragment and $\Psi _X$ is the slowest. The second is a comparison of the fragmentation dynamics in Fig. 4 to the decay of the amplitude of the density oscillations, see Fig. 2. Since fragmentation can develop due to the transverse excitations, also see below, there is no one-to-one correlation between the two properties of the junction, as is the case in one spatial dimension^36,38^. For instance, $\Psi _Y$ is more fragmented than $\Psi _G$, but the density oscillations of the former decay slower than the latter.

Finally, we discuss and compare how the higher natural orbitals become occupied in the four initial states, see Fig. 5. We notice that whenever the initial state is transversely excited, $\Psi _Y$, or is a linear combination consisting of a transversely excited state, $\Psi _V$, it requires a larger number of self-consistent orbitals to accurately represent its dynamical behavior. Furthermore, examining for each state the largest higher natural densities sheds light on the microscopic mechanism of fragmentation. In particular, we find that the second and third natural densities for $\Psi _G$ and the second natural density for $\Psi _X$ have reflection symmetry with no-node in the *y*-direction, they only have excitation in the *x*-direction at $t=10t_{Rabi}$ (not shown). For $\Psi _Y$, we observe that the second, third, and fourth natural densities have one, zero and two nodes at $t=10t_{Rabi}$ in the *y*-direction, respectively (see Fig. S7 of supplemental material). Unlike the other three initial states, the natural densities of the three larger (second, third, and fourth) orbitals for $\Psi _V$ show complex structures having zero, two and one nodes, respectively, in the *x*-*y* plane at $t=10t_{Rabi}$ (see Fig. S8 of supplemental material). However, the shape of the natural orbitals of $\Psi _V$ exhibits the presence of the longitudinal and transverse excitations in the system. Moreover, we notice that the occupancy of all the higher natural orbitals of $\Psi _G$ are essentially identical when calculated in the two-dimensional double-well potential and their corresponding one-dimensional analogs. But from the comparison study for $\Psi _X$, we found that there is a deviation for $\dfrac{n_3(t)}{N}$, with slightly higher occupation in the one-dimensional analog (see Fig. S13 of supplemental material). This deviation signifies that, although the interaction is weak, unlike the survival probability there is no one-to-one correspondence of the development of fragmentation when one moves from two dimensions to one dimension. Rather, the latter instead it depends on the dimensionality of the bosons and how the transverse direction affects the dynamics of a particular initial state.

### Observables and the dynamics of their expectation values and variances

So far, we discussed the transport of the bosons in the junction in terms of the survival probability and the loss of coherence and development of fragmentation in the reduced one-particle density matrix. To shed further light on the time-dependent many-particle wavefunction and on possible geometrical and dimensional effects, we resort to further quantities, the position operator along the *x*- and *y*-direction, the momentum operator along the *x*- and *y*-direction, and the angular-momentum operator of *z* component. Here we demonstrate the dynamics of the expectation values and the variances of the above-mentioned operators and draw a connection with the survival probability and fragmentation.

Let us start with a brief discussion about the expectation values of observables and their dynamics. We find that the expectation value of the ${{\hat{X}}}=\sum _{j=1}^N {{\hat{x}}}_j$ position operator, $\dfrac{1}{N}\langle \Psi |{{\hat{X}}}|\Psi \rangle (t)$, for all initial states possesses a similar structure as found for the respective survival probability profile, see Fig. 2. Namely, at the mean-field level, $\dfrac{1}{N}\langle \Psi |{{\hat{X}}}|\Psi \rangle (t)$ are oscillating in between the two minima of the double-well potential starting from the initial value $-2$ at $t=0$, see Table 1. While at the many-body level, we find numerically that $\dfrac{1}{N}\langle \Psi |{{\hat{X}}}|\Psi \rangle (t)$ for all initial states eventually vanish with time due to the gradual increase of many-body correlations as described in the many-body survival probability (results are not shown).

For $t=0$, we note that the expectation value of the ${{\hat{P}}}_X=\sum _{j=1}^N \frac{1}{i}\frac{\partial }{\partial x_j}$ momentum operator vanishes, $\dfrac{1}{N}\langle \Psi |{{\hat{P}}_X}|\Psi \rangle (0)=0$, due to parity (reflection in *x* for $\Psi _G$, $\Psi _X$, and $\Psi _Y$; inversion through the origin for $\Psi _V$) and translation. Similarly, the expectation values of ${{\hat{Y}}}=\sum _{j=1}^N {{\hat{y}}}_j$ and ${{\hat{P}}}_Y=\sum _{j=1}^N \frac{1}{i}\frac{\partial }{\partial y_j}$ along the transverse direction vanish, $\dfrac{1}{N}\langle \Psi |{{\hat{Y}}}|\Psi \rangle (0)=\dfrac{1}{N}\langle \Psi |{{\hat{P}}_Y}|\Psi \rangle (0)=0$, due to parity (reflection in *y* for $\Psi _G$, $\Psi _X$, and $\Psi _Y$; inversion for $\Psi _V$). Table 1 summarizes the results. At $t>0$ some of these symmetries are exactly conserved, and the expectation values $\dfrac{1}{N}\langle \Psi |{{\hat{Y}}}|\Psi \rangle$ and $\dfrac{1}{N}\langle \Psi |{{\hat{P}}_Y}|\Psi \rangle$ vanish. But the expectation value $\dfrac{1}{N}\langle \Psi |{{\hat{P}}_X}|\Psi \rangle$ shows oscillatory behavior for all states at $t>0$ for the mean-field as well as the many-body dynamics. For $\Psi _G$ and $\Psi _Y$, $\dfrac{1}{N}\langle \Psi |{{\hat{P}}_X}|\Psi \rangle$ keeps on oscillating between $+0.15$ and $-0.15$, while for $\Psi _X$, the values are $+0.70$ and $-0.70$, and for $\Psi _V$, it oscillates between $+0.45$ and $-0.45$ at the mean-field level. The dynamics of $\dfrac{1}{N}\langle \Psi |{{\hat{P}}_X}|\Psi \rangle$ at the many-body level overlaps only initially with the respective expectation values at the mean-field level and eventually shows a decay in amplitude at long-time as many-body correlations develop contrary to the mean-field level.

**Figure 6:**
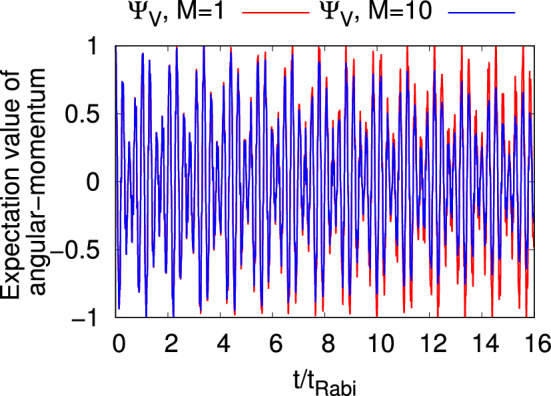
Dynamics of the angular-momentum expectation value per particle, $\dfrac{1}{N}\langle \Psi _V|{{{\hat{L}}_Z}}|\Psi _V\rangle$, in a symmetric 2D double-well for the vortex state $(\Psi _V)$ with $N=10$ bosons. Mean-field ($M=1$ time-adaptive orbitals) result is presented in red and corresponding many-body result ($M=10$ time-adaptive orbitals) in blue. See the text for more details. The quantities shown are dimensionless.

We now move to the angular-momentum which is a fundamental property in 2D taking the operator form ${\hat{L}}_Z=\sum _{j=1}^N \dfrac{1}{i}\left( x_j\dfrac{\partial }{\partial y_j}-y_j\dfrac{\partial }{\partial x_j}\right)$. In connection with the above-discussed quantities, angular-momentum is a combination of position and momentum operators. As is expected, at $t=0$ the expectation value of the angular-momentum operator, $\dfrac{1}{N}\langle \Psi |{{{\hat{L}}_Z}}|\Psi \rangle (0)$, is one for the vortex state while it has a null value for all other states. For $t>0$, $\dfrac{1}{N}\langle \Psi |{{{\hat{L}}_Z}}|\Psi \rangle$ for $\Psi _G$, $\Psi _X$, and $\Psi _Y$ vanishes at the mean-field as well as the many-body level due to the reflection symmetry in *y*. But for $\Psi _V$, $\dfrac{1}{N}\langle \Psi |{{{\hat{L}}_Z}}|\Psi \rangle (t>0)$ shows an interesting oscillatory motion. Recall that angular-momentum is not a conserved quantity in the junction. At the mean-field level this oscillatory behavior is with values between $+1$ and $-1$, where as at the many-body level, there is a decay of the amplitude of oscillations due to the loss of coherence of the vortex state (see Fig 6) and decay of density oscillations (see Fig. S1(d) of supplemental material). Connecting the expectation value of the angular momentum with the density profile of $\Psi _V$, it is found that Fig 3p–y have $\dfrac{1}{N}\langle \Psi |{{{\hat{L}}_Z}}|\Psi \rangle$ values $+1$, 0, $-0.5$, 0, 0.5, 0.5, 0, $-1$, $+1$, and $-0.7$, respectively, at the many-body level. It is noted that the $\dfrac{1}{N}\langle \Psi |{{{\hat{L}}_Z}}|\Psi \rangle$ values at the many-body level initially (until about $t=4t_{Rabi}$) overlap with the corresponding mean-field results (see Fig 6), therefore we observe the same values of $\dfrac{1}{N}\langle \Psi |{{{\hat{L}}_Z}}|\Psi \rangle$ at the mean-field and many-body levels at times when the snapshots of Fig 3(p)-(y) are taken. Figure 3(x) finds the value $+1$ for $\dfrac{1}{N}\langle \Psi |{{{\hat{L}}_Z}}|\Psi \rangle$ even though the density profile does not produce a pure vortex state. This phase difference in the vortex structure occurs due the different tunneling frequency of $\Psi _X$ and $\Psi _Y$. Also, it is noticed that the many-body $\dfrac{1}{N}\langle \Psi |{{{\hat{L}}_Z}}|\Psi \rangle$ is $-0.7$ when the vortex state practically tunnels to the right well (see Fig. 3y).

**Table 1:** Expectation values of the observables and their variances at $t=0$. See the text for more details.

State	$\dfrac{1}{N}\langle {{\hat{X}}}\rangle$	$\dfrac{1}{N}\langle {{\hat{Y}}}\rangle$	$\dfrac{1}{N}\langle {{\hat{P}}_X}\rangle$	$\dfrac{1}{N}\langle {{\hat{P}}_Y}\rangle$	$\dfrac{1}{N}\langle {{\hat{L}}_Z}\rangle$	$\dfrac{1}{N}\Delta _{{{\hat{X}}}}^2$	$\dfrac{1}{N}\Delta _{{{\hat{Y}}}}^2$	$\dfrac{1}{N}\Delta _{{{\hat{P}}_X}}^2$	$\dfrac{1}{N}\Delta _{{{\hat{P}}_Y}}^2$	$\dfrac{1}{N}\Delta _{{{\hat{L}}_Z}}^2$
$\Psi _G$	-2	0	0	0	0	$\dfrac{1}{2}$	$\dfrac{1}{2}$	$\dfrac{1}{2}$	$\dfrac{1}{2}$	2
$\Psi _X$	-2	0	0	0	0	$\dfrac{3}{2}$	$\dfrac{1}{2}$	$\dfrac{3}{2}$	$\dfrac{1}{2}$	3
$\Psi _Y$	-2	0	0	0	0	$\dfrac{1}{2}$	$\dfrac{3}{2}$	$\dfrac{1}{2}$	$\dfrac{3}{2}$	7
$\Psi _V$	-2	0	0	0	1	1	1	1	1	4

The quantities shown are dimensionless.

Now we start the discussion about the many-particle variances of the previously discussed observables at the mean-field and many-body levels. The variances at $t=0$ are analytically calculated and presented in Table 1. At $t=0$, the center-of-mass of the bosonic clouds are at the position $(a,b)=(-2,0)$. To calculate the variances, we have used the general relation between the variances at (*a*, *b*) and at the origin. It is known that for the position and momentum operators, the variances do not change with the position of the center-of-mass of the clouds, i.e., $\dfrac{1}{N}\Delta _{{{\hat{X}}}}^2\Big |_{\Psi (a,b)}=\dfrac{1}{N}\Delta _{{{\hat{X}}}}^2\Big |_{\Psi (0,0)}$, $\dfrac{1}{N}\Delta _{{{\hat{Y}}}}^2\Big |_{\Psi (a,b)}=\dfrac{1}{N}\Delta _{{{\hat{Y}}}}^2\Big |_{\Psi (0,0)}$, $\dfrac{1}{N}\Delta _{{{\hat{P}}_X}}^2\Big |_{\Psi (a,b)}=\dfrac{1}{N}\Delta _{{{\hat{P}}_X}}^2\Big |_{\Psi (0,0)}$, and $\dfrac{1}{N}\Delta _{{{\hat{P}}_Y}}^2\Big |_{\Psi (a,b)}=\dfrac{1}{N}\Delta _{{{\hat{P}}_Y}}^2\Big |_{\Psi (0,0)}$. For our system, the variance of the angular-momentum operator boils down to (see supplemental material and^80^)

4 $$\begin{aligned} \dfrac{1}{N}\Delta _{{{\hat{L}}_Z}}^2\Big |_{\Psi (a,b)}=\dfrac{1}{N}\Delta _{{{\hat{L}}_Z}}^2\Big |_{\Psi (0,0)}+a^2\dfrac{1}{N}\Delta _{{{\hat{P}}_Y}}^2\Big |_{\Psi (0,0)}. \end{aligned}$$

**Figure 7:**
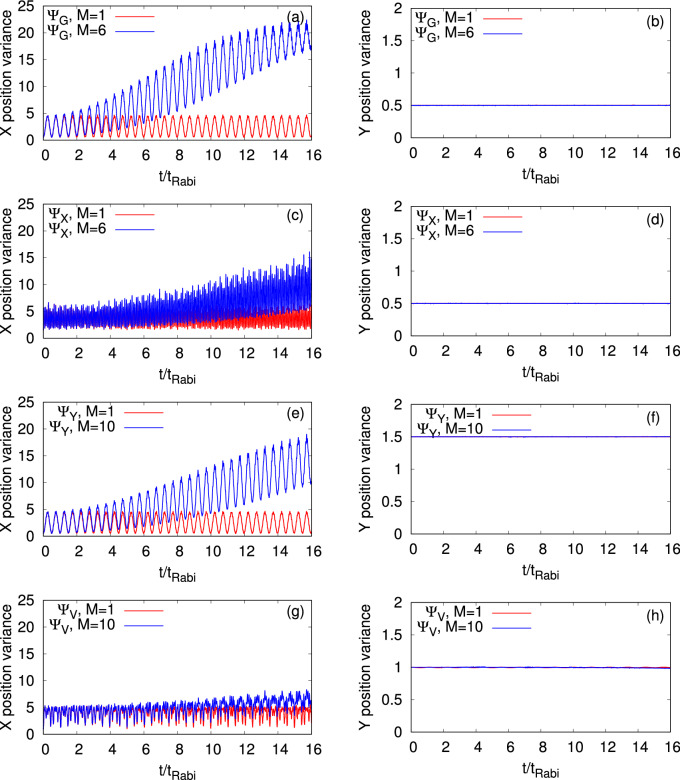
The mean-field ($M=1$ time-adaptive orbitals, in red) and many-body (in blue) time-dependent position variances per particle, $\dfrac{1}{N}\Delta _{{\hat{X}}}^2(t)$ and $\dfrac{1}{N}\Delta _{{\hat{Y}}}^2(t)$, are presented in the left and right columns, respectively. The different initial states, $\Psi _G$, $\Psi _X$, $\Psi _Y$, and $\Psi _V$, for $N=10$ bosons are plotted row-wise. The many-body results are computed using the MCTDHB method with $M=6$ orbitals for $\Psi _{G}$ and $\Psi _{X}$ and $M=10$ orbitals for $\Psi _{Y}$ and $\Psi _{V}$. See the text for more details. The quantities shown are dimensionless.

**Figure 8:**
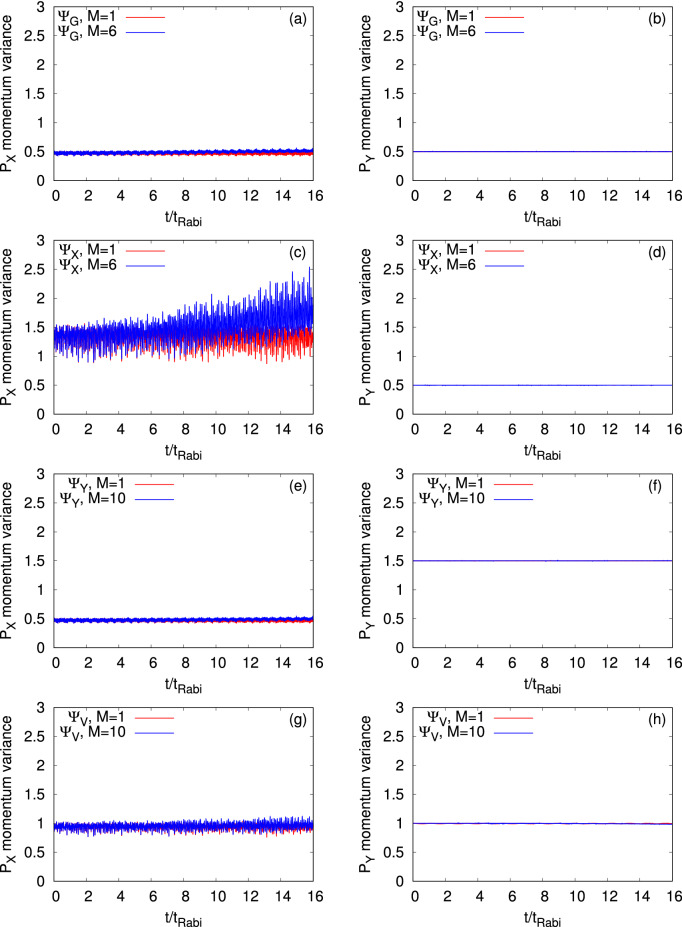
The mean-field ($M=1$ time-adaptive orbitals, in red) and many-body (in blue) time-dependent position variances per particle, $\dfrac{1}{N}\Delta _{{{\hat{P}}_X}}^2(t)$ and $\dfrac{1}{N}\Delta _{{{\hat{P}}_Y}}^2(t)$, are presented in the left and right columns, respectively. The different initial states, $\Psi _G$, $\Psi _X$, $\Psi _Y$, and $\Psi _V$, for $N=10$ bosons are plotted row-wise. The many-body results are computed using the MCTDHB method with $M=6$ orbitals for $\Psi _{G}$ and $\Psi _{X}$ and $M=10$ orbitals for $\Psi _{Y}$ and $\Psi _{V}$. See the text for more details. The quantities shown are dimensionless.

**Figure 9:**
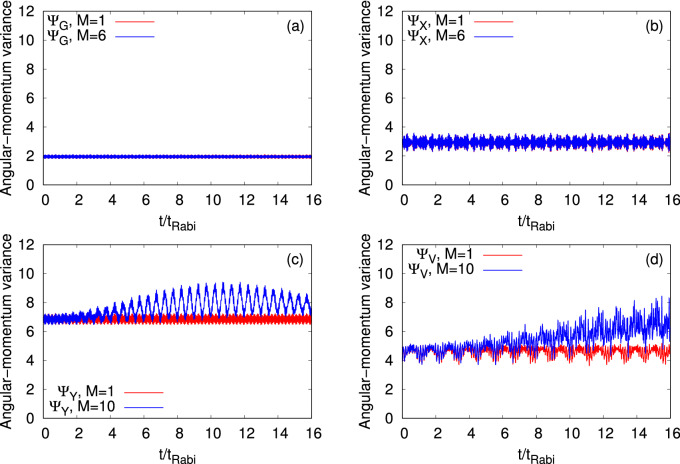
Dynamics of the angular-momentum variance per particle, $\dfrac{1}{N}\Delta _{{{\hat{L}}_Z}}^2(t)$, in a symmetric 2D double-well for the initial states (**a**) $\Psi _G$, (**b**) $\Psi _X$, (**c**) $\Psi _Y$, and (**d**) $\Psi _V$ with $N=10$ bosons. The interaction parameter $\Lambda =0.01\pi$. Mean-field ($M=1$ time-adaptive orbitals) results are presented in red and corresponding many-body results are shown in blue. MCTDHB results are computed with $M=6$ time-adaptive orbitals for $\Psi _{G}$ and $\Psi _{X}$, and $M=10$ time-adaptive orbitals for $\Psi _{Y}$ and $\Psi _{V}$. See the text for more details. The quantities shown are dimensionless.

In Fig. 7, we present the time-dependent many-particle position variances per particle, $\dfrac{1}{N}\Delta _{{\hat{X}}}^2(t)$ and $\dfrac{1}{N}\Delta _{{\hat{Y}}}^2(t)$, in a symmetric 2D double-well potential for all the initial states of the bosonic clouds. We show that the many-body correlations can lead to a deviation in $\dfrac{1}{N}\Delta _{{\hat{X}}}^2(t)$ which can not be seen at the mean-field level. Both the many-body and mean-field values of $\dfrac{1}{N}\Delta _{{\hat{X}}}^2(t)$ vary in time in an oscillatory manner for all the initial states considered here with the highest frequency of oscillations for $\Psi _X$ which is consistent with the respective survival probability. However, there are couple of clear differences that can be seen due to the many-body correlations. Here the mean-field $\dfrac{1}{N}\Delta _{{\hat{X}}}^2(t)$ oscillates with a constant amplitude, whereas the many-body $\dfrac{1}{N}\Delta _{{\hat{X}}}^2(t)$ oscillates with a growing amplitude. Moreover, the pace of growth of $\dfrac{1}{N}\Delta _{{\hat{X}}}^2(t)$ is different for different initial states. One of the interesting features shown in Fig. 7 is that the minima values of the many-body $\dfrac{1}{N}\Delta _{{\hat{X}}}^2(t)$ increase with time for each of the initial states with a maximal deviation occurs for $\Psi _G$. The increase of minima values of $\dfrac{1}{N}\Delta _{{\hat{X}}}^2(t)$ due to the growing degree of fragmentation can be found in the literature but only for the ground state in one-dimensional double-well potentials^39,40^. Also, we notice high-frequency small-amplitude oscillations, specially for the vortex state, on top of the peaks of the large-amplitude oscillations of $\dfrac{1}{N}\Delta _{{\hat{X}}}^2(t)$. Such high-frequency oscillations occur due to the breathing-mode oscillations of the system. As $\Psi _G$ and $\Psi _X$ have one-dimensional analogs, we compare $\dfrac{1}{N}\Delta _{{\hat{X}}}^2(t)$ for these states obtained from the two- and one-dimensional double-wells both at the mean-field and many-body levels, and we find a consistency between $\dfrac{1}{N}\Delta _{{\hat{X}}}^2(t)$ and fragmentation dynamics. The comparison shows that only the many-body $\dfrac{1}{N}\Delta _{{\hat{X}}}^2(t)$ of $\Psi _X$ have a small difference between two dimensions and corresponding one dimension analog (see Fig. S14 of supplemental material). It suggests that the impact of the transverse direction on different quantities are different for different initial states.

Unlike $\dfrac{1}{N}\Delta _{{\hat{X}}}^2(t)$, the mean-field and many-body values of $\dfrac{1}{N}\Delta _{{\hat{Y}}}^2(t)$ have very small fluctuations, of the order of $10^{-3}$, and therefore their dynamics look more of a constant at the presented scale. The overlap of the mean-field and many-body values of $\dfrac{1}{N}\Delta _{{\hat{Y}}}^2(t)$ tells us that the mean-field results are a good approximation of the many-body results for the position variance in the transverse direction. $\dfrac{1}{N}\Delta _{{\hat{Y}}}^2(t)$ suggests that even though there is practically no motion along the *y*-direction, the combination of the motion along the *x*-direction with the existence of the almost-frozen transverse degree of freedom could lead to dynamics of the angular-momentum variance in the junction, as will be shown below, which can not be accounted in the one-dimensional geometry.

To show whether the many-body correlations have any effect on the variance of momentum operator, we compare the many-body $\dfrac{1}{N}\Delta _{{{\hat{P}}_X}}^2(t)$ and $\dfrac{1}{N}\Delta _{{{\hat{P}}_Y}}^2(t)$ with the corresponding mean-field results. Here it is worthwhile to mention that the momentum variance is comparatively a more complex quantity than the position variance in the junction as the former one is more sensitive to changes in the shape of the orbitals. In Fig. 8, we see that the mean-field $\dfrac{1}{N}\Delta _{{{\hat{P}}_X}}^2(t)$ oscillates around a certain value for each of the initial states considered here. But the many-body $\dfrac{1}{N}\Delta _{{{\hat{P}}_X}}^2(t)$ shows oscillations with a slowly growing values. It is found that $\dfrac{1}{N}\Delta _{{{\hat{P}}_X}}^2(t)$ for $\Psi _X$ are always higher than for the other states. We observe that $\dfrac{1}{N}\Delta _{{{\hat{P}}_X}}^2(t)$ for $\Psi _V$ is in between the respective results of $\Psi _X$ and $\Psi _Y$ till the time considered here, which is more evident in the momentum variance along the *y*-direction, discussed below. The high frequency oscillations occurring in the momentum variance, which are more prominent for the states $\Psi _X$ and $\Psi _V$, are due to the stronger breathing oscillations of the system along the *x*-direction. As discussed in case of $\dfrac{1}{N}\Delta _{{\hat{X}}}^2(t)$, we also compare $\dfrac{1}{N}\Delta _{{{\hat{P}}_X}}^2(t)$ of $\Psi _G$ and $\Psi _X$ between their two dimensions and corresponding one dimension analogs and observe a small difference only for the many-body $\dfrac{1}{N}\Delta _{{{\hat{P}}_X}}^2(t)$ of $\Psi _X$ (see Fig. S15 of supplemental material).

As presented in the discussion of $\dfrac{1}{N}\Delta _{{\hat{Y}}}^2(t)$, the momentum variance along the *y*-direction, $\dfrac{1}{N}\Delta _{{{\hat{P}}_Y}}^2(t),$ also exhibits very small fluctuations, of the order of $10^{-3}$, for all the initial states of bosonic clouds both at the mean-field as well as the many-body level. The mean-field and many-body values of $\dfrac{1}{N}\Delta _{{{\hat{P}}_Y}}^2(t)$ practically overlap with each other with almost constant values, being 0.5, 0.5, 1.5, and 1.0 for the states $\Psi _G$, $\Psi _X$, $\Psi _Y$, and $\Psi _V$, respectively. As the vortex state is the combination of $\Psi _X$ and $\Psi _Y$, $\dfrac{1}{N}\Delta _{{{\hat{P}}_Y}}^2(t)$ for $\Psi _V$ is exactly in between the corresponding results of $\Psi _X$ and $\Psi _Y$. Similarly to $\dfrac{1}{N}\Delta _{{\hat{Y}}}^2(t)$, the non-zero values of $\dfrac{1}{N}\Delta _{{{\hat{P}}_Y}}^2(t)$ with small fluctuations indicate the existence of transverse motion of the bosonic clouds which will have a consequential effect to the dynamics of the angular-momentum variance in the junction.

Now, we move to the discussion of the angular-momentum variance per particle, $\dfrac{1}{N}\Delta _{{{\hat{L}}_Z}}^2(t)$, presented in Fig. 9. We observe a marginal difference in the angular-momentum variance calculated at the many-body and mean-field levels for the states $\Psi _G$ and $\Psi _X$, implying that the mean-field theory will be enough to discuss $\dfrac{1}{N}\Delta _{{{\hat{L}}_Z}}^2(t)$ for these two states. Fig. 9a finds that $\dfrac{1}{N}\Delta _{{{\hat{L}}_Z}}^2(t)$ of $\Psi _G$ oscillates with amplitude of fluctuations in the order of $10^{-1}$. In comparison with $\Psi _G$, $\Psi _X$ has a larger amplitude of oscillations of $\dfrac{1}{N}\Delta _{{{\hat{L}}_Z}}^2(t)$ which varies from the value 2.4 to 3.6. The fluctuations in the angular-momentum at the many-body level are governed by the structure of the many-body wave-function, shapes of the time-dependent orbitals, and the mechanism of fragmentation. Therefore, without excitation in the *y*-direction, fragmentation occurring due to the barrier in the *x*-direction does hardly impact the fluctuations in the angular-momentum for $\Psi _G$ and $\Psi _X$. From Fig. 9a,b, one can find that the amount of fluctuations atop the base-line are practically same at the mean-field and many-body levels. These fluctuations are around $5\%$ and $20\%$ for the states $\Psi _G$ and $\Psi _X$, respectively.

Contrary to $\Psi _G$ and $\Psi _X$, exciting many-body features have been found for the angular-momentum variance of $\Psi _Y$ and $\Psi _V$. Figure 9c,d show that the many-body $\dfrac{1}{N}\Delta _{{{\hat{L}}_Z}}^2(t)$ for $\Psi _Y$ and $\Psi _V$ are oscillatory in nature with a growing amplitude. Also, their minima values are increasing with time. The maximal fluctuations on top of the baseline of the angular-momentum variance for $\Psi _Y$ and $\Psi _V$ at the mean-field level are found around $7\%$ and $25\%$, respectively, while at the many-body level they are approximately $34\%$ and $112\%$, respectively. A difference in the onset of the angular-momentum fluctuations for $\Psi _Y$ and $\Psi _V$ at the many-body level can be described by analyzing how fragmentation develops in the system. Unlike $\Psi _G$ and $\Psi _X$, transverse excitation is involved in the lowest excited natural orbitals, $\phi _2$, $\phi _3$, and $\phi _4$ (see Figs. S7 and S8 in the supplemental material) of $\Psi _Y$ and $\Psi _V$, leading to large fluctuations in the angular-momentum variance. It is found that after $t\approx 12 t_{\text {Rabi}}$, the amplitude of the oscillations of $\dfrac{1}{N}\Delta _{{{\hat{L}}_Z}}^2(t)$ starts decaying for $\Psi _Y$. $\dfrac{1}{N}\Delta _{{{\hat{L}}_Z}}^2(t)$ of $\Psi _V$ shows two types of oscillations very prominently, one with a larger amplitude and smaller frequency which arises due to the density oscillations and the second one with a smaller amplitude but higher frequency due to the breathing oscillations of the system. Another feature of $\dfrac{1}{N}\Delta _{{{\hat{L}}_Z}}^2(t)$ is that the mean-field values of $\dfrac{1}{N}\Delta _{{{\hat{L}}_Z}}^2(t)$ for $\Psi _Y$ are always larger than the respective values for $\Psi _V$, but the many-body $\dfrac{1}{N}\Delta _{{{\hat{L}}_Z}}^2(t)$ of $\Psi _V$ eventually becomes in time larger compared to the corresponding values of $\Psi _Y$. The features of all the quantum mechanical observables, expectation values, and their variances discussed above certainly determine that the mean-field level of theory is not sufficient to accurately explain the dynamics of a trapped system in a two-dimensional geometry.

### Long-time dynamics

**Figure 10:**
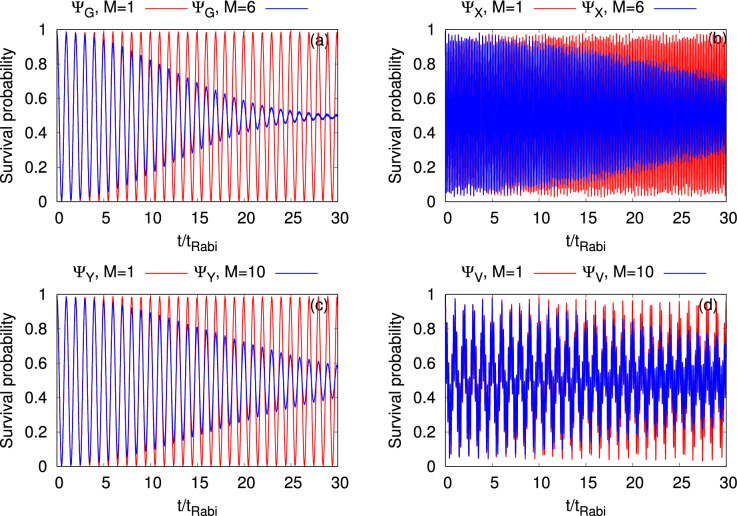
Long-time dynamical evolution of the survival probability of $N=10$ bosons in the left well of a symmetric 2D double-well for the initial states (**a**) $\Psi _G$, (**b**) $\Psi _X$, (**c**) $\Psi _Y$, and (**d**) $\Psi _V$. The interaction parameter $\Lambda =0.01\pi$. Mean-field results (in red) and corresponding many-body results (in blue). MCTDHB results are computed with $M=6$ time-adaptive orbitals for $\Psi _{G}$ and $\Psi _{X}$, and $M=10$ time-adaptive orbitals for $\Psi _{Y}$ and $\Psi _{V}$. The quantities shown are dimensionless.

**Figure 11:**
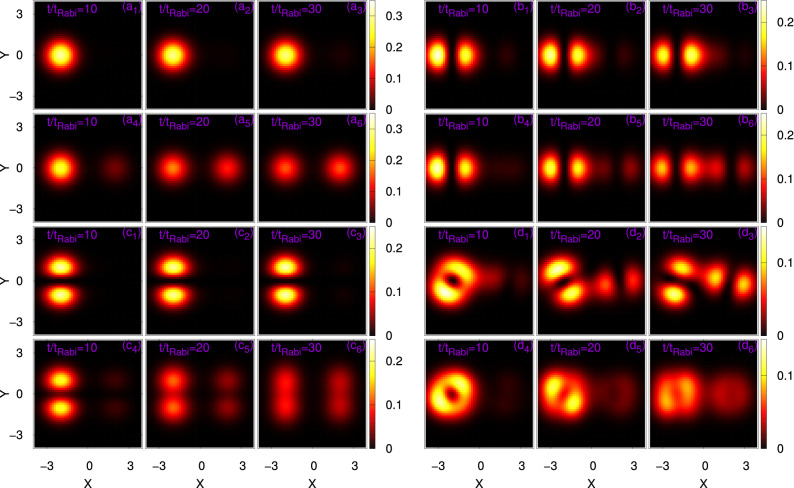
Time evolution of the mean-field (first and third rows) and many-body (second and fourth rows) density oscillations in a symmetric 2D double-well. The interaction parameter is $\Lambda =0.01\pi$ and the number of bosons is $N=10$. Shown are snapshots for the densities per particle at $t=10t_{Rabi}$ (first and fourth columns), $20t_{Rabi}$ (second and fifth columns), and $30t_{Rabi}$ (third and sixth columns) for the initial states $\Psi _G$, $\Psi _X$, $\Psi _Y$, and $\Psi _V$. The MCTDHB computation is performed with $M=6$ time-adaptive orbitals for $\Psi _{G}$ and $\Psi _{X}$, and with $M=10$ time-adaptive orbitals for $\Psi _{Y}$ and $\Psi _{V}$. The quantities shown are dimensionless.

So far, we have displayed in a detail study the dynamical behavior of the density oscillations, loss of coherence, development of fragmentation, expectation values, and variances of a few basic quantum mechanical operators in a symmetric 2D double-well in the short to intermediate time domain $$(t=0$$ to $$16t_{Rabi})$$. The results show that the presence of transverse excitations requires a larger number of time adaptive orbitals to accurately represent the many-body effects of the quantities discussed here. Before ending this section, it is worthwhile to include a flavour of the long-time dynamics of the most basic property, $$P_L(t)$$. In Fig. 10, we have registered the long-time dynamics of $$P_L(t)$$ for the four initial states $$\Psi _G$$, $$\Psi _X$$, $$\Psi _Y$$, and $$\Psi _V$$. Snapshots of the density oscillations are shown in Fig. 11. The long-time dynamics of other quantities along with their convergence are discussed in the supplemental material. The plots show that the densities of the systems tunnel back and forth without changing the amplitude and frequency at the mean-field level, even for the long-time dynamics. One can clearly observe that the many-body $$P_L(t)$$ displays a collapse in the oscillations, for all the initial bosonic clouds. The collapse of the oscillations is already shown only for the ground state, both in theoretically^38^ and experimentally^38^. However, we find that rate of collapse is different for different initial states. Among the four initial states, the collapse of $$\Psi _G$$ is the quickest and of $$\Psi _V$$ is the slowest for this symmetric 2D double-well.

The collapse of the initial states are consistent with the density oscillations shown in Fig. 11. The snapshots of the mean-field and many-body density oscillations are taken at $t=10t_{Rabi}$, $20t_{Rabi}$, and $30t_{Rabi}$. Unlike the dynamics at the mean-field level, the many-body dynamics of $\Psi _G$, $\Psi _X$, and $\Psi _Y$ show the generation of replicas of the respective initial states in the process of tunneling. But the time evolution of the vortex state in a double-well is completely different in comparison with the other three initial states. Both at the mean-field and many-body levels, $\Psi _V$ generates two dipoles at the two potential minima and they change their orientation in the process of evolution. It can be seen from the figure that in spite of the generation of vortex dipoles in two dissimilar level of theory, the evolution of the dipoles are different in terms of shape and orientation due to the development of the many-body fragmentation in the system.

## Concluding remarks

In the present work, we have studied the tunneling dynamics of initially coherent bosonic clouds in a two-dimensional double-well potential. The bosonic systems are prepared either as the ground, transversely or longitudinally excited, or vortex state in the left well of a symmetric 2D double-well potential. Although, the tunneling dynamics of the ground and longitudinally excited states have one-dimensional manifestations, here we examine their two-dimensional analogs by solving the full many-body Schrödinger equation and compare the results with their one-dimensional analogs. Moreover, we study the transversely excited and vortex states which do not have any one-dimensional analog, and require at least a two-dimensional geometry to be realized.

Explicitly, we have performed the numerical simulations to study the dynamics of the $\Psi _G$, $\Psi _X$, $\Psi _Y$, and $\Psi _V$ states based on a well-known method, MCTDHB. We observe the dynamical behavior of a few physical quantities such as the survival probability in the left well, depletion and fragmentation, and the many-particle position, momentum, and angular-momentum expectation values and variances of each of the bosonic clouds when they tunnel back and forth in the double-well potential. To show the impact of growing degree of fragmentation with time, we compare the respective quantities at the many-body level of theory with their respective mean-field results.

We have shown that apart from the vortex state, all other initial states can tunnel through the barrier without destroying their initial structures at the mean-field level as well as at the many-body level. But the vortex state distorts its structure while it tunnels and produces two vortex dipoles which rotate around the minima of the corresponding well both at the mean-field and many-body levels. However, the shape and orientation of the dipoles, in the long-time dynamics, are found to be different at the mean-field level in comparison with the many-body level when one observes the density oscillations of $\Psi _V$. We find that the creation of the vortex dipoles occurs due to differences in the tunneling frequencies of $\Psi _X$ and $\Psi _Y$. Moreover, the effect of the many-body correlation appears even in the dynamics of the most basic quantity, i.e., the survival probability, in terms of the collapse of the density oscillations which can not be seen using the Gross–Pitaevskii theory. Also, the collapse rates are found to be different for the different initial states considered here. Therefore, the loss of coherence and development of fragmentation demonstrate the clear signature of many-body correlations on the dynamics of the bosonic clouds in the two-dimensional BJJ. We notice that the rate of loss of coherence or development of fragmentation is maximum for $\Psi _Y$ and minimum for $\Psi _X$. Examining the first few natural orbitals for all the states, we observe that the presence of transverse excitations enhances the loss of coherence for $\Psi _Y$ and $\Psi _V$, and impacts the dynamics of physical quantities discussed in this work. Interestingly, the increase of loss of coherence due to the transverse excitations occurs even when the transverse degrees-of-freedom as quantified by $\dfrac{1}{N}\Delta _{{\hat{Y}}}^2(t)$ and $\dfrac{1}{N}\Delta _{{\hat{P}}_Y}^2(t)$ are seemingly frozen, see below.

Based on the time-evolution of the survival probability and fragmentation, we have further discussed how the many-body correlations affect some basic quantum mechanical observables and their variances. Precisely, we present the interconnection of many-particle variances, $\dfrac{1}{N}\Delta _{{\hat{X}}}^2(t)$, $\dfrac{1}{N}\Delta _{{\hat{Y}}}^2(t)$, $\dfrac{1}{N}\Delta _{{\hat{P}}_X}^2(t)$, $\dfrac{1}{N}\Delta _{{\hat{P}}_Y}^2(t)$, and $\dfrac{1}{N}\Delta _{{\hat{L}}_Z}^2(t)$ with the density oscillations and fragmentation. The many-body variances incorporate the depletion and fragmentation, generally leading to different values with the respective mean-field variances. It is observed that the time-evolution of each variance vary due to the different initial structures of the bosonic cloud. The distinctive feature of the breathing-mode oscillations in addition to the density oscillations are found in the time evolution of $\dfrac{1}{N}\Delta _{{\hat{X}}}^2(t)$, $\dfrac{1}{N}\Delta _{{\hat{P}}_X}^2(t)$, and $\dfrac{1}{N}\Delta _{{\hat{L}}_Z}^2(t)$. The breathing-mode oscillations are the most prominent for the initial states $\Psi _X$ and $\Psi _V$. We show that the existence of the transverse degrees-of-freedom, although nearly frozen, can have significant influence on angular-momentum properties in the system. It is clear from the investigation that the information of the many-body features can not be extracted from only the shape of the density profile of the system, but it requires a close analysis of the natural orbitals and microscopic mechanism of the fragmentation. The present investigation shows that the tunneling dynamics of the ground, excited, and vortex states in two-dimension bosonic Josephson junction is very rich and many-body theory is required to accurately represent their dynamics. We believe that our work will motivate researchers to study the out-of-equilibrium tunneling dynamics of more complex and intricate objects.

## Supplementary information

Supplementary Information
