# Supplementary material for: Impact of the transverse direction on the many-body tunneling dynamics in a two-dimensional bosonic Josephson junction
Source: Sci Rep. 2020 Dec 8;10:21476. doi: 10.1038/s41598-020-78173-w (PMC7722723; doi:10.1038/s41598-020-78173-w)
Supplement: Supplementary file 1 — Supplementary Information [file 41598_2020_78173_MOESM1_ESM.pdf]

Supplemental material for Impact of the transverse direction on the many-body tunneling dynamics in a two-dimensional bosonic Josephson junction

Anal Bhowmik,[1,2,*] Sudip Kumar Haldar,[1,2,3] and Ofir E. Alon[1,2]

[1] *Department of Mathematics, University of Haifa, Haifa 3498838, Israel*

[2] *Haifa Research Center for Theoretical Physics and Astrophysics, University of Haifa, Haifa 3498838, Israel*

[3] *Department of Physics, SRM University Delhi-NCR, Plot No. 39 Rajiv Gandhi education city, Sonipat 131029, India*

[*] abhowmik@campus.haifa.ac.il

In this supplemental material, we augment the main text with further details. In Section 1, we present a brief mathematical description on the many-particle variance discussed in the main text. In Section 2, we show the long-time dynamics of the quantum mechanical quantities along with their numerical convergence with respect to the number of time-adaptive orbitals and discrete-variable-representation grid points. In Section 3, we show the consistency of the preparation of the ground state. As there are one-dimensional analogs for the ground and longitudinally-excited states, in Section 4, we include a derivation to move from two dimensions to one dimension along with a detailed comparison study between the results obtained from the two-dimensional double-well and their one-dimensional analogs.

I. MANY-PARTICLE VARIANCE

The quantum variance of an observable $\hat{A}$ for a system in a state $|\Psi(t)\rangle$ determines the quantum resolution with which the observable can be measured. The variance of $\hat{A}$ is measured by the combination of the expectation values of $\hat{A}$ and the square of $\hat{A}$. Here the expectation value of $\hat{A} = \sum_{j=1}^{N} \hat{a}(r_j)$ is solely made of one-body operators but the expectation of the square of $\hat{A}$, $\hat{A}^2 = \sum_{j=1}^{N} \hat{a}^2(r_j) + \sum_{j<k} 2\hat{a}(r_j)\hat{a}(r_k)$, is a mixture of one- and two-body operators. The variance can be expressed as [1]

$$\begin{aligned}\frac{1}{N}\Delta^2_{\hat{A}}(t) &= \frac{1}{N}[\langle\Psi(t)|\hat{A}^2|\Psi(t)\rangle - \langle\Psi(t)|\hat{A}|\Psi(t)\rangle^2] \\ &= \frac{1}{N}\Bigg\{\sum_j n_j(t)\int d\mathbf{r}\phi_j^*(\mathbf{r};t)\hat{a}^2(\mathbf{r})\phi_j(\mathbf{r};t) - \left[\sum_j n_j(t)\int d\mathbf{r}\phi_j^*(\mathbf{r};t)\hat{a}(\mathbf{r})\phi_j(\mathbf{r};t)\right]^2 \\ &+ \sum_{jpkq}\rho_{jpkq}(t)\left[\int d\mathbf{r}\phi_j^*(\mathbf{r};t)\hat{a}(\mathbf{r})\phi_k(\mathbf{r};t)\right]\left[\int d\mathbf{r}\phi_p^*(\mathbf{r};t)\hat{a}(\mathbf{r})\phi_q(\mathbf{r};t)\right]\Bigg\}, \end{aligned} \tag{1.1}$$

where $\{\phi_j(\mathbf{r};t)\}$ are the natural orbitals, $\{n_j(t)\}$ the natural occupations, and $\rho_{jpkq}(t)$ are the elements of the reduced two-particle density matrix, $\rho(\mathbf{r}_1,\mathbf{r}_2,\mathbf{r}_1',\mathbf{r}_2';t) = \sum_{jpkq}\rho_{jpkq}(t)\phi_j^*(\mathbf{r}_1';t)\phi_p^*(\mathbf{r}_2';t)$ $\phi_k(\mathbf{r}_1;t)\phi_q(\mathbf{r}_2;t)$. For one-body operators which are local in position space, the variance described in Eq 1.1 becomes [2]

$$\begin{aligned}\frac{1}{N}\Delta^2_{\hat{A}}(t) &= \int d\mathbf{r}\frac{\rho(\mathbf{r};t)}{N}\hat{a}^2(\mathbf{r}) - N\left[\int\frac{\rho(\mathbf{r};t)}{N}\hat{a}(\mathbf{r})\right]^2 \\ &+ \int d\mathbf{r}_1 d\mathbf{r}_2\frac{\rho^{(2)}(\mathbf{r}_1,\mathbf{r}_2,\mathbf{r}_1,\mathbf{r}_2;t)}{N}a(\mathbf{r}_1)a(\mathbf{r}_2). \end{aligned} \tag{1.2}$$

In our study, the center-of-mass of the bosonic clouds are at the position $(a, b) = (-2, 0)$ at $t = 0$. Eq 1.1 describes the variances when the center-of-mass of the bosonic clouds are at (0, 0). To calculate the variances at (a, b), we have used the general relation between the variances at (a, b) and at the origin. As mentioned in the main text, for the position and momentum operators, the variances do not change with the position of center-of-mass of the clouds [1], i.e., $\frac{1}{N}\Delta^2_{\hat{X}}\Big|_{\Psi(a,b)} = \frac{1}{N}\Delta^2_{\hat{X}}\Big|_{\Psi(0,0)}$, $\frac{1}{N}\Delta^2_{\hat{Y}}\Big|_{\Psi(a,b)} = \frac{1}{N}\Delta^2_{\hat{Y}}\Big|_{\Psi(0,0)}$, $\frac{1}{N}\Delta^2_{\hat{P}_X}\Big|_{\Psi(a,b)} = \frac{1}{N}\Delta^2_{\hat{P}_X}\Big|_{\Psi(0,0)}$, and $\frac{1}{N}\Delta^2_{\hat{P}_Y}\Big|_{\Psi(a,b)} = \frac{1}{N}\Delta^2_{\hat{P}_Y}\Big|_{\Psi(0,0)}$. But the situation becomes a more involved for the variance of the angular-momentum operator, which takes the form as [1]

$$\begin{aligned}
\frac{1}{N}\Delta^2_{\hat{L}_Z}\Big|_{\Psi(a,b)} &= \frac{1}{N}\Delta^2_{\hat{L}_Z}\Big|_{\Psi(0,0)} + \frac{1}{N}a^2\Delta^2_{\hat{P}_Y}\Big|_{\Psi(0,0)} + \frac{1}{N}b^2\Delta^2_{\hat{P}_X}\Big|_{\Psi(0,0)} \\
&+ \frac{1}{N}\Big\{a[\langle\Psi(0,0)|\hat{L}_Z\hat{P}_Y + \hat{P}_Y\hat{L}_Z|\Psi(0,0)\rangle - 2\langle\Psi(0,0)|\hat{L}_Z|\Psi(0,0)\rangle\langle|\Psi(0,0)|\hat{P}_Y|\Psi(0,0)\rangle] \\
&- b[\langle\Psi(0,0)|\hat{L}_Z\hat{P}_X + \hat{P}_X\hat{L}_Z|\Psi(0,0)\rangle - 2\langle\Psi(0,0)|\hat{L}_Z|\Psi(0,0)\rangle\langle|\Psi(0,0)|\hat{P}_X|\Psi(0,0)\rangle] \\
&- 2ab[\langle\Psi(0,0)|\hat{P}_Y\hat{P}_X|\Psi(0,0)\rangle - \langle\Psi(0,0)|\hat{P}_Y|\Psi(0,0)\rangle\langle|\Psi(0,0)|\hat{P}_X|\Psi(0,0)\rangle]\Big\}. \qquad (1.3)
\end{aligned}$$

Because of symmetry, Eq. 1.3 boils down to $\frac{1}{N}\Delta^2_{\hat{L}_Z}\Big|_{\Psi(a,b)} = \frac{1}{N}\Delta^2_{\hat{L}_Z}\Big|_{\Psi(0,0)} + a^2\frac{1}{N}\Delta^2_{\hat{P}_Y}\Big|_{\Psi(0,0)}$ for our considered systems in this work (see the subsection 3.3 in the main text). The final form of $\frac{1}{N}\Delta^2_{\hat{L}_Z}\Big|_{\Psi(a,b)}$ is used directly to calculate the angular-momentum variance at $t = 0$, see Table 1 of the main text.

II. LONG-TIME DYNAMICS AND CONVERGENCE OF QUANTITIES

Here we check the numerical convergence for the long-time dynamics of our results discussed in the main text with respect to the number of time-adaptive orbitals and density of the grid points for the ground (Ψ_G), longitudinally-excited (Ψ_X), transversely-excited (Ψ_Y), and vortex (Ψ_V) states.

A. Convergence with the number of time-adaptive orbitals

In our work, we have performed the computations for the Ψ_G and Ψ_X states with $M = 6$ time-adaptive orbitals, while for the Ψ_Y and Ψ_V states using $M = 10$ time-adaptive orbitals. To check the convergence with the orbital numbers, we repeat our computations with $M = 10$ and $M = 12$ orbitals for Ψ_G, Ψ_X and Ψ_Y, Ψ_V, respectively. As discussed in the main text, we have prepared the initial states of the bosonic clouds in the left well of a symmetric double-well with

$N = 10$ bosons. The interaction parameter is $\Lambda = 0.01\pi$, also see Section 3 below. The many-body Hamiltonian is represented by 64×64 exponential discrete-variable-representation grid points in a box size $[-10, 10) \times [-10, 10)$.

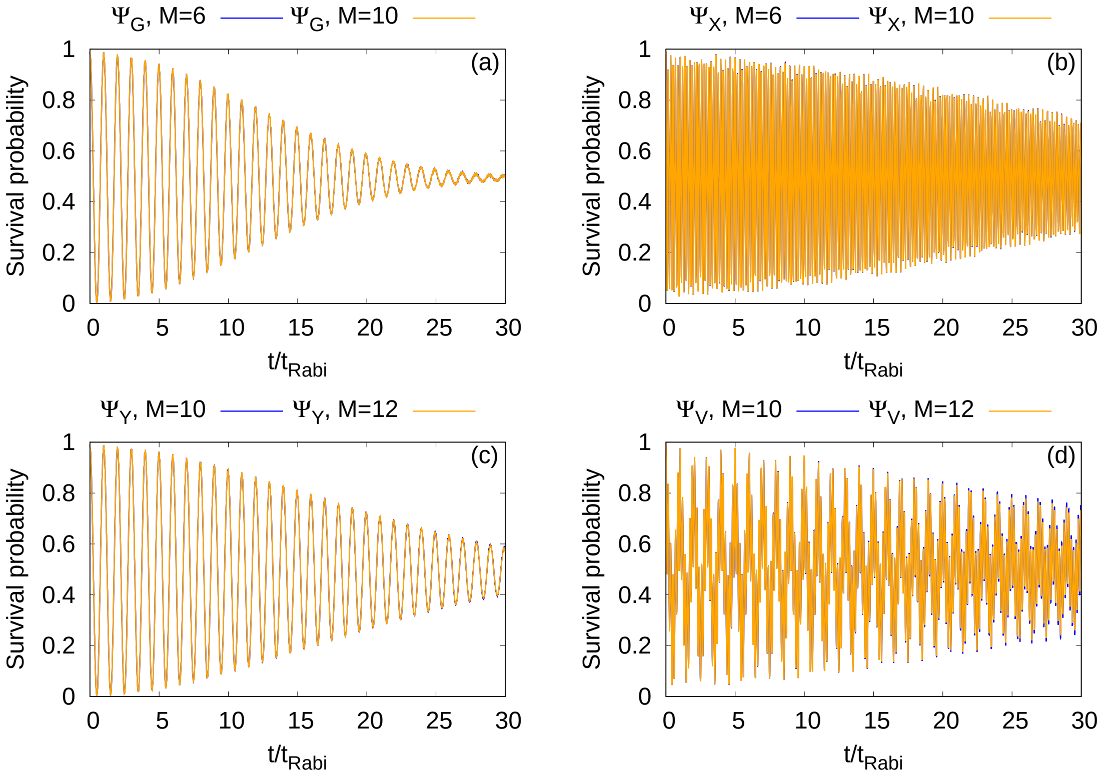

FIG. S1. Convergence of the survival probability, $P_L(t)$, with respect to the number of time-adaptive orbitals for the initial states (a) Ψ_G, (b) Ψ_X, (c) Ψ_Y, and (d) Ψ_V of $N = 10$ interacting bosons with $\Lambda = 0.01\pi$ in the symmetric double-well trap. The many-body results are computed using the MCTDHB method. The convergence are verified with $M = 6$, 10 time-adaptive orbitals for the states, Ψ_G and Ψ_X. While we demonstrate the convergence of the results for Ψ_Y and Ψ_V using $M = 10$, 12 time-adaptive orbitals. The quantities shown are dimensionless.

We demonstrate the numerical convergence with the orbital numbers of the many-particle $P_L(t)$, $\frac{1}{N}\Delta^2_{\hat{X}}(t)$, $\frac{1}{N}\Delta^2_{\hat{P}_X}(t)$, $\frac{1}{N}\langle\Psi_V|\hat{L}_Z|\Psi_V\rangle$, and $\frac{1}{N}\Delta^2_{\hat{L}_Z}(t)$ in Figs. S1, S2, S3, S4, and S5, respectively. The variances of the position and momentum operators along the y-direction for all states have very small fluctuations (of the order of 10^{-3}) with a function of time, and they practically overlap with the corresponding mean-field results (see subsection "Observables and the dynamics of their expectation values and variances" in the main text). Therefore, we have not shown explicitly the convergences of variances of the position and momentum operators along the y-direction in our presentation which, of course, converge as well.

As discussed in the main text, the collapse of $P_L(t)$ is prominent in the the long-time dynamics

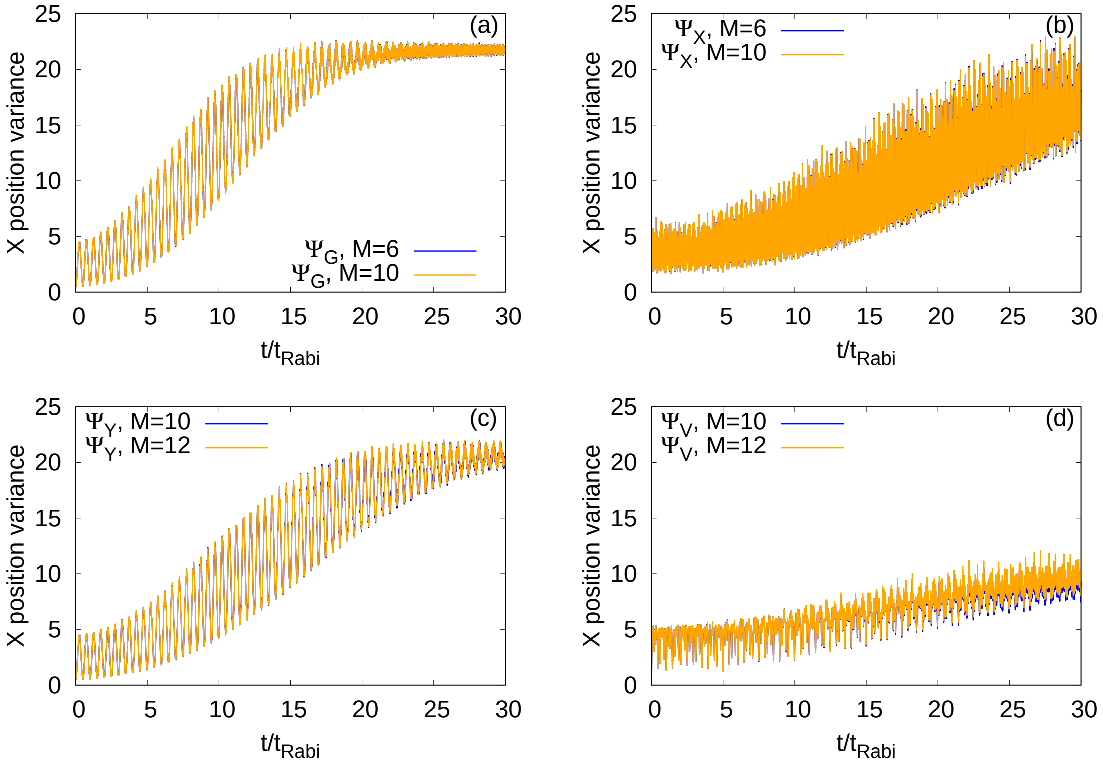

FIG. S2. Convergence of the time-dependent many-body position variance per particle along x-direction, $\frac{1}{N}\Delta^2_{\hat{X}}(t)$, in a symmetric double-well trap with the number of time-adaptive orbitals for the initial states (a) Ψ_G, (b) Ψ_X, (c) Ψ_Y, and (d) Ψ_V for $N = 10$ interacting bosons with $\Lambda = 0.01\pi$. The many-body $\frac{1}{N}\Delta^2_{\hat{X}}(t)$ are computed using the MCTDHB method. The convergence are verified with $M = 6$, 10 time-adaptive orbitals for the states, Ψ_G and Ψ_X. While we demonstrate the convergence of the results for Ψ_Y and Ψ_V using $M = 10$, 12 time-adaptive orbitals. See the text for more details. The quantities shown are dimensionless.

for all initial states. Here also we observe the collapse in the overall oscillation of $P_L(t)$ when computed with $M = 10$ and $M = 12$ time adaptive orbitals for Ψ_G, Ψ_X and Ψ_Y, Ψ_V, respectively. For all initial states, $P_L(t)$ show a complete overlap when their respective time-adaptive orbital numbers are increased indicating that the dynamics of $P_L(t)$ is already converged for $M = 6$ and $M = 10$ orbitals for Ψ_G, Ψ_X and Ψ_Y, Ψ_V, respectively. We verify that the small amplitude and high frequency oscillations of $P_L(t)$ for Ψ_V computed using $M = 10$ and 12 time-adaptive orbitals fall on top of each other.

The long-time dynamics of $\frac{1}{N}\Delta^2_{\hat{X}}(t)$ for all initial states and their convergence with the number of time-adaptive orbitals are presented in Fig. S2. The dynamics of $\frac{1}{N}\Delta^2_{\hat{X}}(t)$ obtained from larger number of time-adaptive orbitals falls on top of the respective variances with smaller number of time-adaptive orbitals exhibiting the convergence with the orbital numbers. The effect of increased

degree of the fragmentation can be seen for each of the initial states as discussed in the main text. The consequences of the density oscillations and breathing mode oscillations (occurred due to the effect of coupling between the lowest energy band and the higher excited states) are also being observed in the long-time dynamics in terms of two kind of oscillations, i.e., small frequency with large amplitude and high frequency with small amplitude oscillations. A noticeable difference in the long-time dynamics is that $\frac{1}{N}\Delta^2_{\hat{X}}(t)$ reaches its equilibrium which is more evident for Ψ_G and Ψ_Y. This equilibration-like effect comes to the picture if the density oscillations collapse and the fragmentation reaches its plateau. Therefore, here we find a consistent behavior of $\frac{1}{N}\Delta^2_{\hat{X}}(t)$ with the survival probability and fragmentation also for an excited state like Ψ_Y.

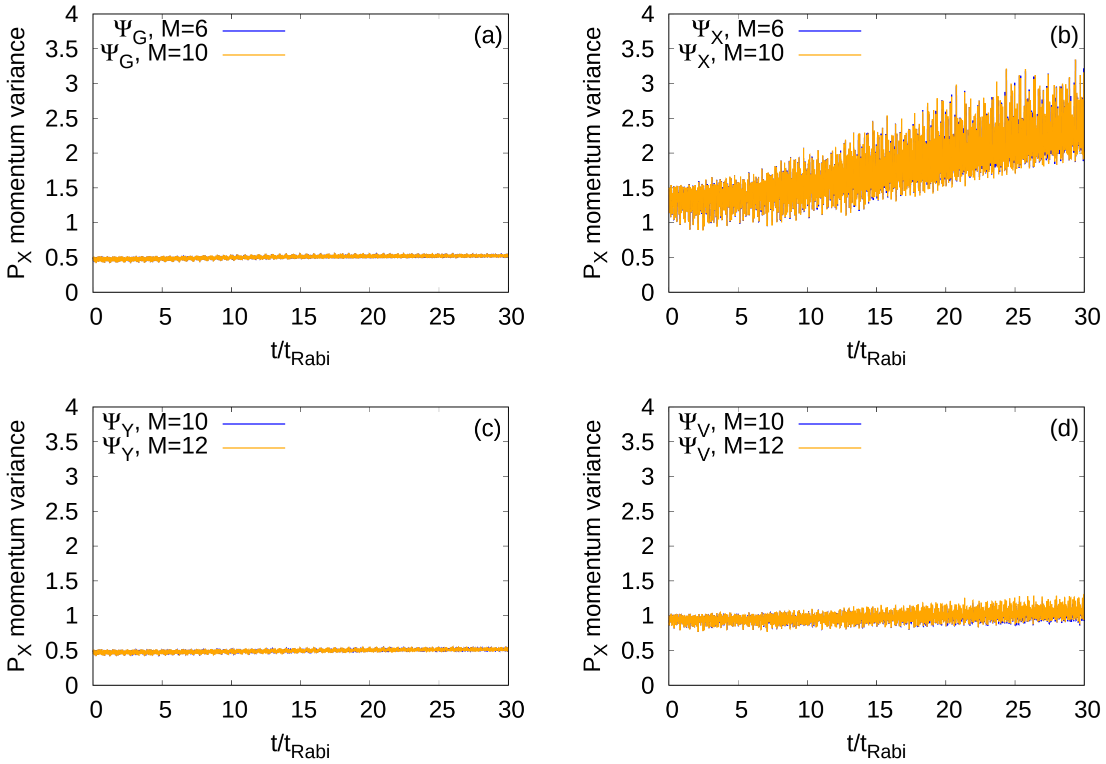

FIG. S3. Convergence of the time-dependent many-body momentum variance per particle along x-direction, $\frac{1}{N}\Delta^2_{\hat{P}_X}(t)$, in a symmetric double-well trap with the number of time-adaptive orbitals for the initial states (a) Ψ_G, (b) Ψ_X, (c) Ψ_Y, and (d) Ψ_V for $N = 10$ interacting bosons with $\Lambda = 0.01\pi$. The many-body $\frac{1}{N}\Delta^2_{\hat{P}_X}(t)$ are computed using the MCTDHB method. The convergence are verified with $M = 6$, 10 time-adaptive orbitals for the states, Ψ_G and Ψ_X. While we demonstrate the convergence of the results for Ψ_Y and Ψ_V using $M = 10$, 12 time-adaptive orbitals. See the text for more details. The quantities shown are dimensionless.

Similar to $\frac{1}{N}\Delta^2_{\hat{X}}(t)$, we find that the converged results of $\frac{1}{N}\Delta^2_{\hat{P}_X}(t)$ with the orbital numbers,

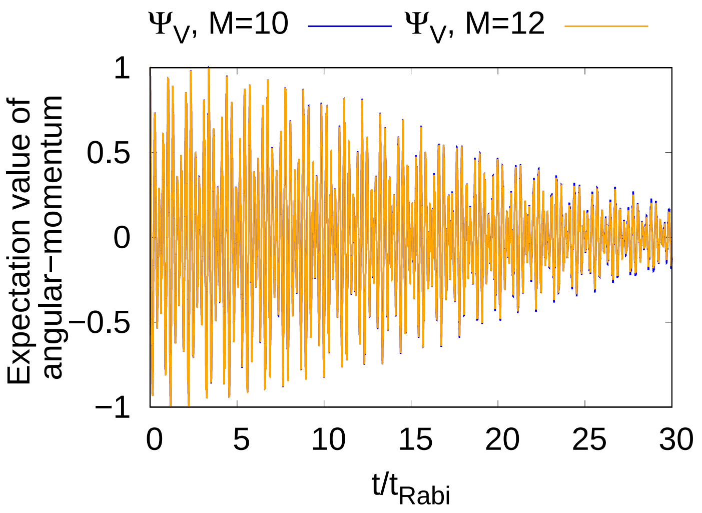

FIG. S4. Long-time dynamics of the angular-momentum expectation value per particle, $\frac{1}{N}\langle\Psi_V|\hat{L}_Z|\Psi_V\rangle$, in a symmetric double-well for the vortex state, Ψ_V. The number of bosons is $N = 10$. The interaction parameter is $\Lambda = 0.01\pi$. The convergence is verified with $M = 10$ and 12 time-adaptive orbitals. The mean-field $\frac{1}{N}\langle\Psi_V|\hat{L}_Z|\Psi_V\rangle$ would not produce any collapse (not shown) as presented here (see Fig. 6 in the main text). See the text for more details. The quantities shown are dimensionless.

see Fig. S3. $\frac{1}{N}\Delta^2_{\hat{P}_X}(t)$ of Ψ_G and Ψ_Y keep on fluctuating with a smaller amplitude with respect to Ψ_X and Ψ_V even in the long-time dynamics. The presence of the breathing mode oscillations in the dynamics of $\frac{1}{N}\Delta^2_{\hat{P}_X}(t)$ is prominent for Ψ_X and Ψ_V. A comparative study of $\frac{1}{N}\Delta^2_{\hat{P}_X}(t)$ for all initial states shows that $\frac{1}{N}\Delta^2_{\hat{P}_X}(t)$ for Ψ_X is the most influenced by the many-body effect, see Fig. 8 of the main text.

In Fig. S4, we demonstrate the convergence with the orbital number of $\frac{1}{N}\langle\Psi|\hat{L}_Z|\Psi\rangle$ for the vortex state. The main text shows the beginning of the collapse of $\frac{1}{N}\langle\Psi|\hat{L}_Z|\Psi\rangle$ due to the many-body correlations, which continues in the long-time dynamics presented here. It shows that the average angular-momentum tends to zero in the many-body long-time dynamics for a vortex state. By comparing Fig S1 (d) and Fig S4, it is found that the decay of $\frac{1}{N}\langle\Psi|\hat{L}_Z|\Psi\rangle$ is faster than that of $P_L(t)$ for the vortex state.

Fig. S5 presents the long-time dynamics of the many-body variance of the most sensitive quantum mechanical observable presented in this work, i.e. $\frac{1}{N}\Delta^2_{\hat{L}_Z}(t)$. Simultaneously, the figure ensures the convergence with the orbital numbers for each of the initial state. As mentioned in the dynamics of $\frac{1}{N}\Delta^2_{\hat{P}_X}(t)$, here also we observe a prominent breathing oscillations accompanied by density oscillations. For Ψ_G and Ψ_X, fluctuations of $\frac{1}{N}\Delta^2_{\hat{L}_Z}(t)$ continue their trend in the long-time

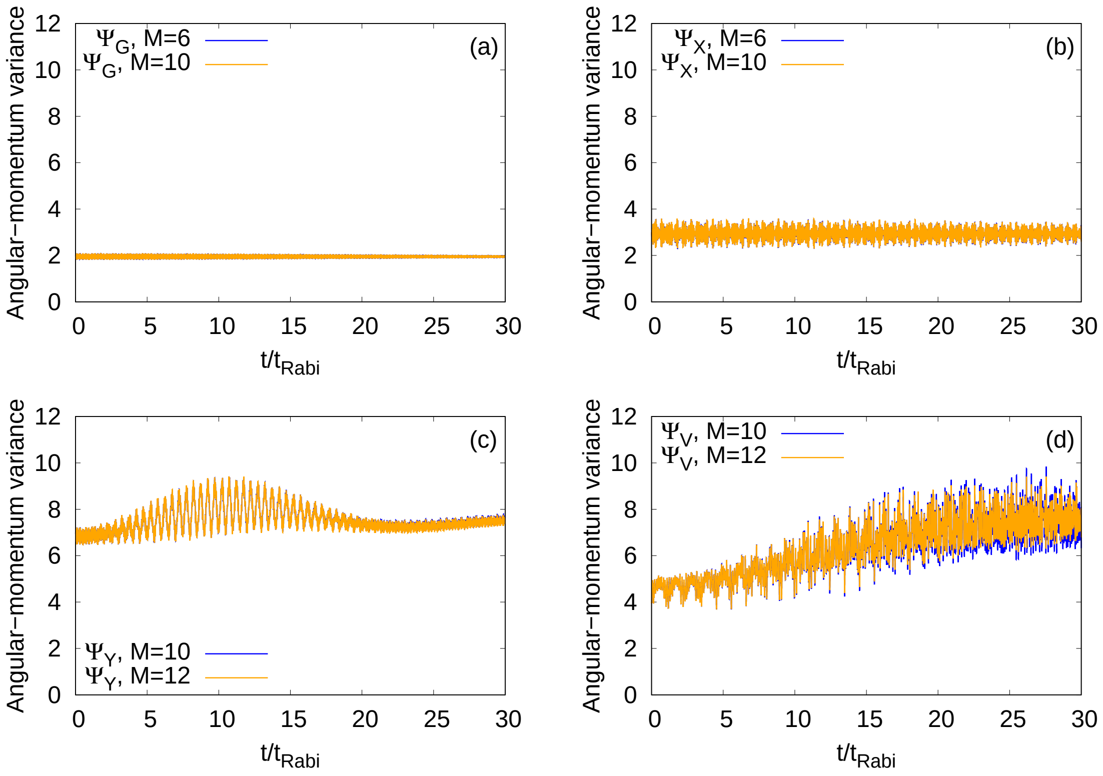

FIG. S5. Convergence of the time-dependent many-body angular-momentum variance per particle, $\frac{1}{N}\Delta^2_{\hat{L}_Z}(t)$), in a symmetric double-well trap with the number of time-adaptive orbitals for the initial states (a) Ψ_G, (b) Ψ_X, (c) Ψ_Y, and (d) Ψ_V for $N = 10$ interacting bosons with $\Lambda = 0.01\pi$. The many-body $\frac{1}{N}\Delta^2_{\hat{P}_X}(t)$ are computed using the MCTDHB method. The convergence are verified with $M = 6$, 10 time-adaptive orbitals for the states, Ψ_G and Ψ_X. While we demonstrate the convergence of the results for Ψ_Y and Ψ_V using $M = 10$, 12 time-adaptive orbitals. See the text for more details. The quantities shown are dimensionless.

dynamics as observed in the short-time dynamics. But for Ψ_Y and Ψ_V, the many-body $\frac{1}{N}\Delta^2_{\hat{L}_Z}(t)$ almost reach an equilibration in the long-time dynamics after the short-time growth dynamics.

In Fig. S6, we plot the natural occupancy of the orbitals per particle, $\frac{n_j(t)}{N}$, for the four initial states, Ψ_G, Ψ_X, Ψ_Y, and Ψ_V. Results are obtained with $M = 6$, 10 time-adaptive orbitals for Ψ_G and Ψ_X, and $M = 10$, 12 time-adaptive orbitals for Ψ_Y and Ψ_V. We find that the results for Ψ_G and Ψ_X with $M = 6$ and $M = 10$ completely fall on top of each other for the two largest occupation numbers, $\frac{n_1(t)}{N}$ and $\frac{n_2(t)}{N}$. The natural occupations $\frac{n_3(t)}{N}$ to $\frac{n_6(t)}{N}$ of Ψ_G and Ψ_X are very small in magnitude (less than 10^{-3}) and almost completely overlap in comparison when computed using $M = 6$ and 10 time-adaptive orbitals. In case of Ψ_Y, the first eight orbitals are showing fully converged results with the number of orbitals. Small deviations can be observed

for $\frac{n_9(t)}{N}$ and $\frac{n_{10}(t)}{N}$ of Ψ_Y when one compares the results, computed from $M = 10$ and 12 time-adaptive orbitals. The latter exhibit in comparison very small occupations (less than 10^{-3}). Finally, as shown in the figure, the occupations of all the natural orbitals of Ψ_V are well converged with the number of orbitals. The results signify that the fragmentation dynamics of all objects studied in the present work are well converged and whenever the transverse excitations exist in the system, more natural orbitals are required to represent the dynamics accurately.

We discussed in the main text that the largest natural orbitals of Ψ_G and Ψ_X show only excitations in the x-direction, the direction along which the barrier is formed, with no-node in the y-direction. Therefore, here we discuss the first four highest natural orbitals of Ψ_Y and Ψ_V, as their occupancies are greater than 10^{-1} in the long-time dynamics. The results at $t = 10t_{Rabi}$, $20t_{Rabi}$ and $30t_{Rabi}$ for Ψ_Y and Ψ_V are presented in Fig. S7 and S8, respectively. For Ψ_Y, the 1st and 4th natural orbitals look like the 1st and 2nd excited states in y, respectively. Interestingly, the 2nd and 3rd natural orbitals of Ψ_Y show a change in order in time. The natural orbitals of Ψ_V show comparatively complex structures as they have the combined effect of Ψ_X and Ψ_Y. The 1st and 2nd natural orbitals of Ψ_V have one and zero nodes in the x-y plane, respectively. Similar to Ψ_Y, we find that there is a change in order of natural orbitals for Ψ_V in time but this happen between the 3rd and 4th natural orbitals.

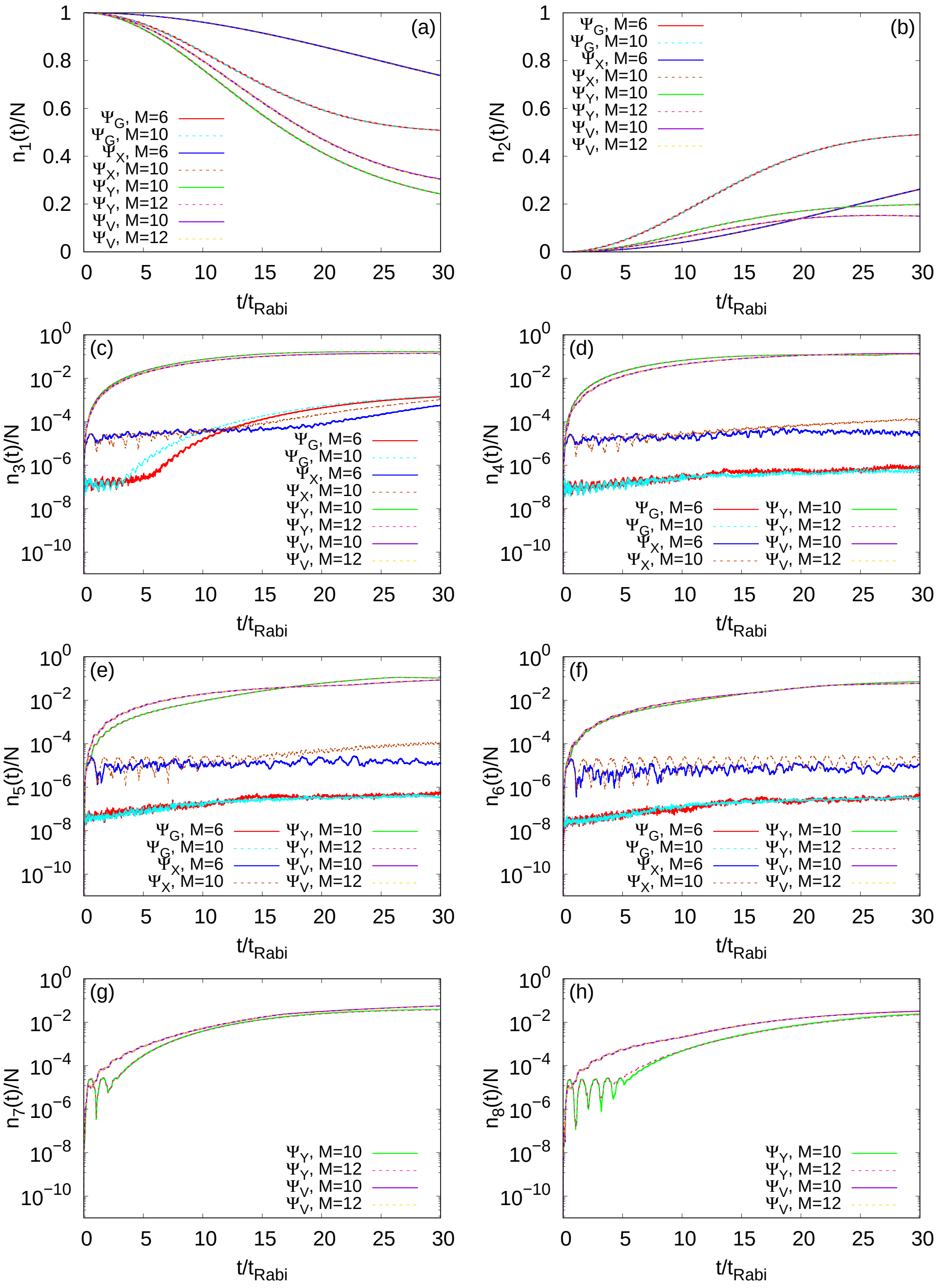

FIG. S6. Continued

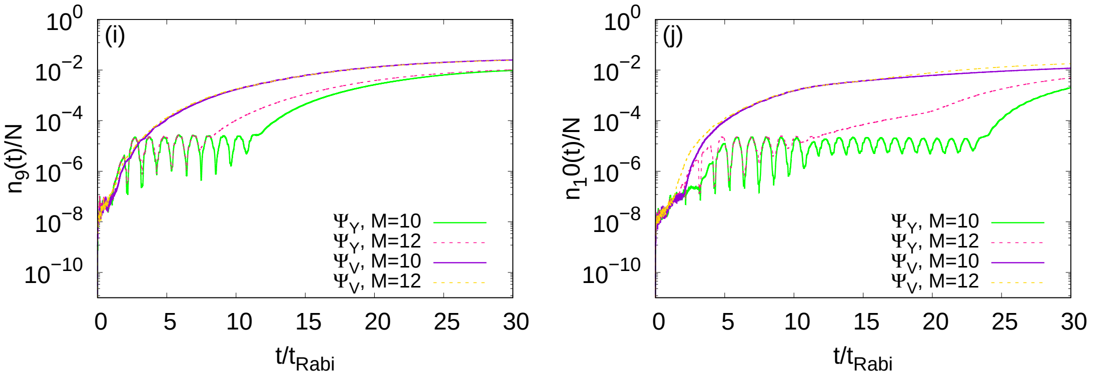

FIG. S6. Convergence of the natural occupation numbers per particle, $\frac{n_j(t)}{N}$, as a function of time with the number of time-adaptive orbitals for the initial states Ψ_G, Ψ_X, Ψ_Y, and Ψ_V in the symmetric 2D double-well trap. The number of bosons is $N = 10$. The interaction parameter is $\Lambda = 0.01\pi$. The many-body results are computed using the MCTDHB method. The convergence are verified with $M = 6$, 10 time-adaptive orbitals for the states, Ψ_G and Ψ_X. While we demonstrate the convergence of the results for Ψ_Y and Ψ_V using $M = 10$, 12 time-adaptive orbitals. Convergence of the time-dependent occupation number from top to bottom (largest to smallest) is demonstrated for all bosonic clouds. The plots reveal that at long propagation times a large number of self-consistent orbitals are needed to accurately represent the tunneling dynamics of the considered states. Color codes are explained in each panel. See the text for more details. The quantities shown are dimensionless.

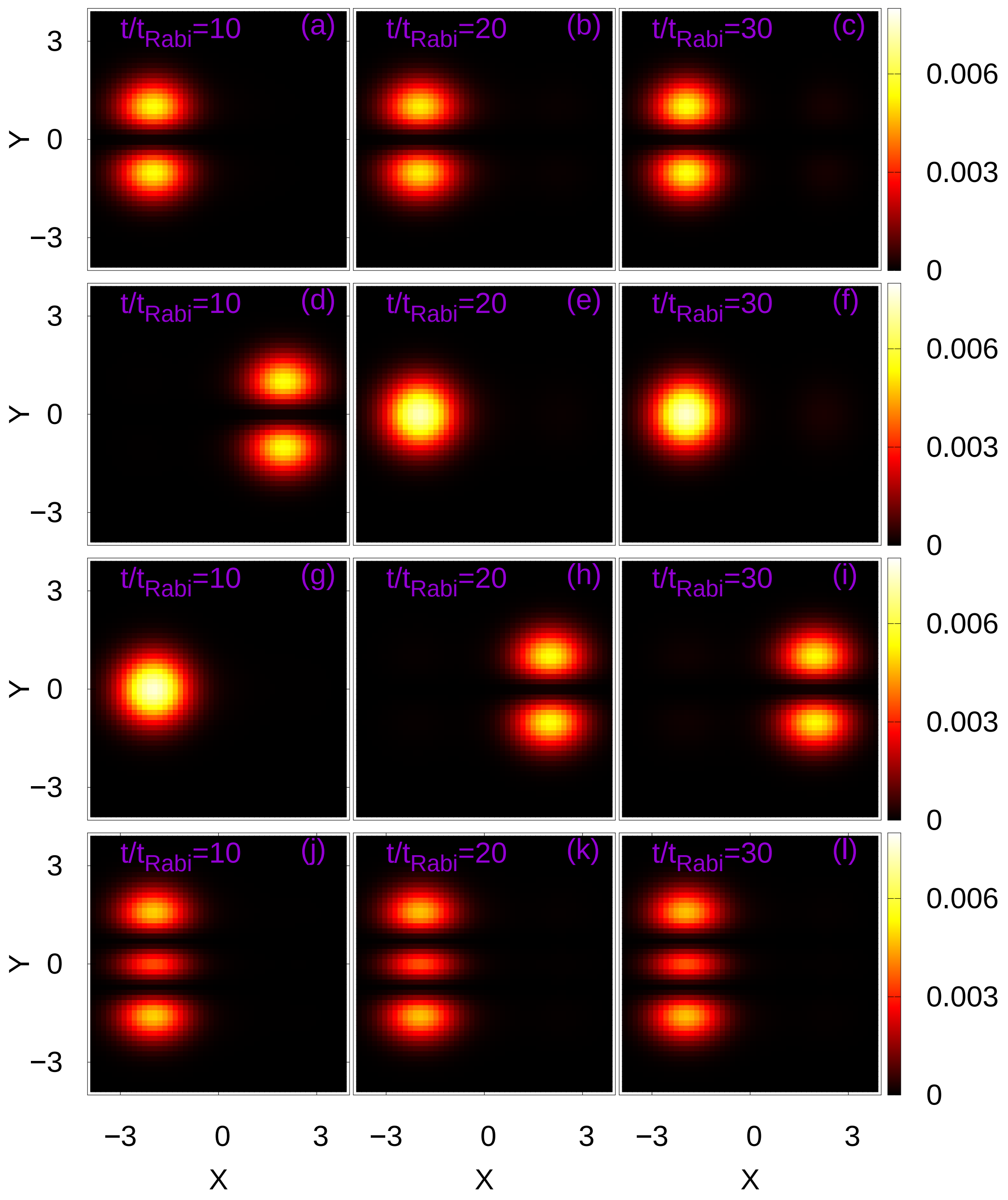

FIG. S7. Time evolution of the natural orbital densities, $|\phi_j(\mathbf{r})|^2$ where j = 1, 2, 3, and 4 (row wise), in a symmetric 2D double-well for Ψ_Y. The interaction parameter is $\Lambda = 0.01\pi$ and the number of bosons is $N = 10$. The MCTDHB computation is performed with with $M = 10$ time-adaptive orbitals. Shown are snapshots at $t = 10t_{Rabi}$ (first column), $20t_{Rabi}$ (second column), and $30t_{Rabi}$ (third column). See the text for more details. The quantities shown are dimensionless.

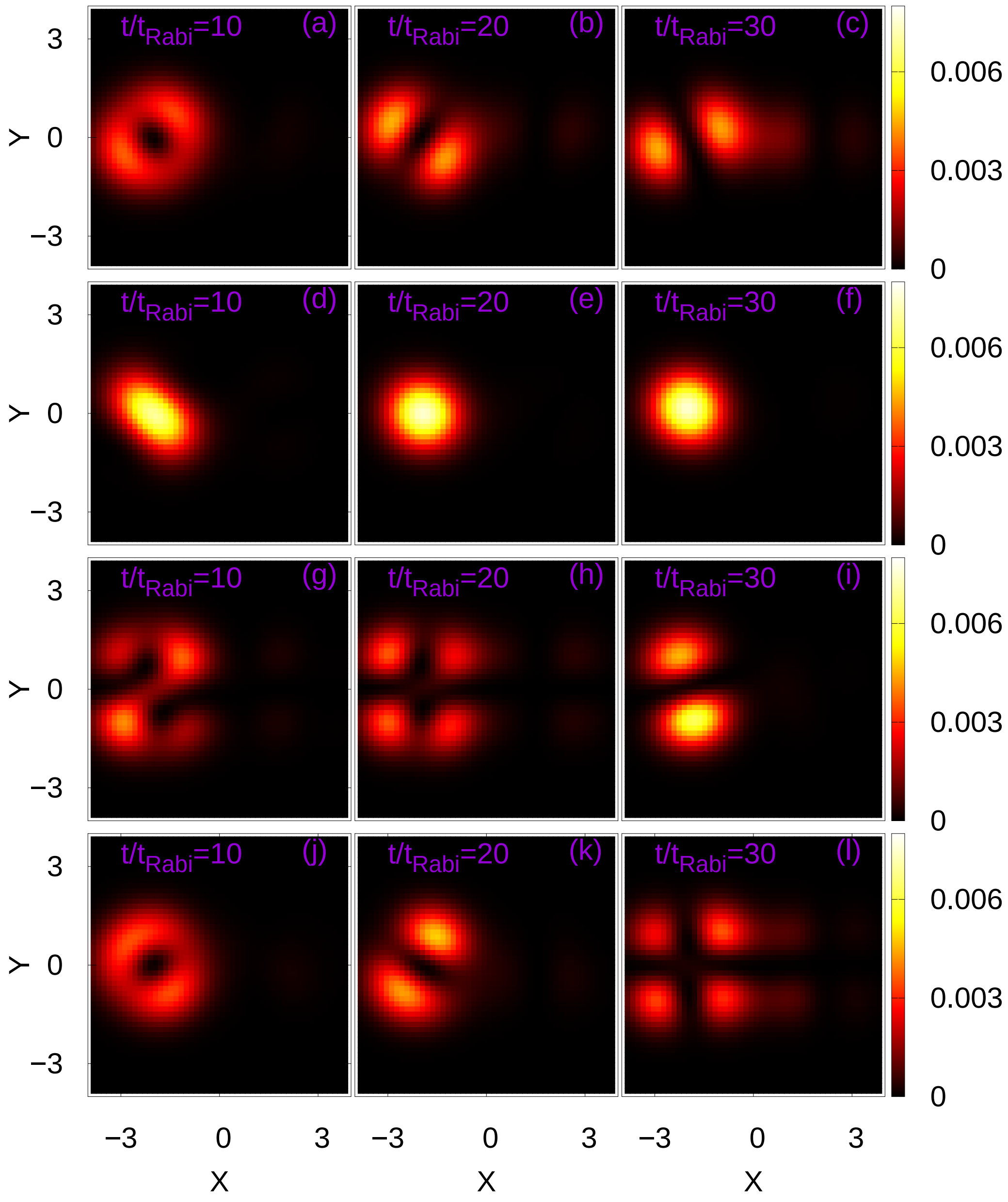

FIG. S8. Time evolution of the natural orbital densities, $|\phi_j(\mathbf{r})|^2$ where $j = 1$, 2, 3, and 4 (row wise), in a symmetric 2D double-well for Ψ_V. The interaction parameter is $\Lambda = 0.01\pi$ and the number of bosons is $N = 10$. The MCTDHB computation is performed with with $M = 10$ time-adaptive orbitals. Shown are snapshots at $t = 10t_{Rabi}$ (first column), $20t_{Rabi}$ (second column), and $30t_{Rabi}$ (third column). See the text for more details. The quantities shown are dimensionless.

B. Convergence with the number of grid points

In the main text, we have computed all quantities, survival probability, fragmentation, expectation value, and variance, with 64×64 grid points. In order to verify the convergence with the grid points, we repeat our computation with 128×128 grid points for all objects. To demonstrate the convergence with the grid points, we choose two many-body variances which have high sensitivity, $\frac{1}{N}\Delta^2_{\hat{X}}(t)$ and $\frac{1}{N}\Delta^2_{\hat{L}_Z}(t)$ of the vortex state and plot the results. Fig. S9 exhibits that increasing the density of the grid points does not have any visible effect on the results presented in this work which signifies the convergence with the number of grid points.

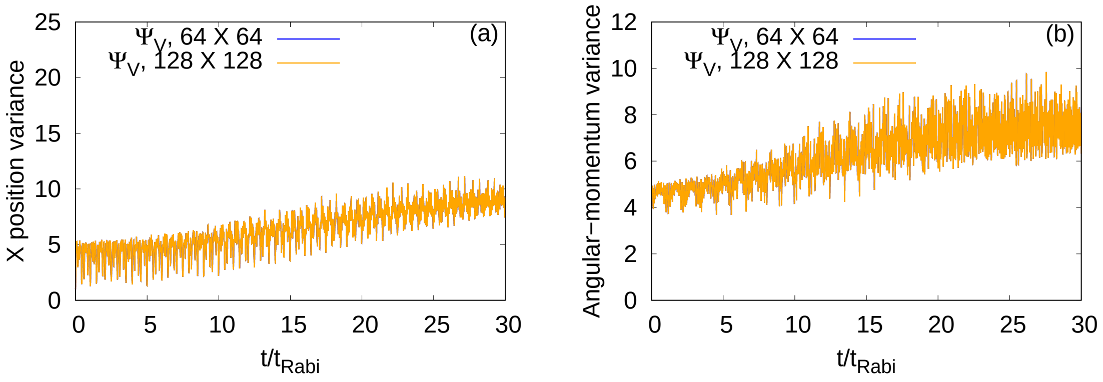

FIG. S9. Convergence of the (a) time-dependent many-body position variance per particle along the x-direction, $\frac{1}{N}\Delta^2_{\hat{X}}(t)$ and (b) many-body angular-momentum variance per particle, $\frac{1}{N}\Delta^2_{\hat{L}_Z}(t)$, in a symmetric 2D double-well trap with the number of grid points for the vortex state. The number of interacting bosons is $N = 10$. The interaction parameter is $\Lambda = 0.01\pi$. The many-body results are computed using the MCTDHB method. The convergence are demonstrated with 64×64 and 128×128 grid points. See the text for more details. The quantities shown are dimensionless.

III. CONSISTENCY OF THE INITIAL-STATE PREPARATION FOR THE DYNAMICS

Here we check the consistency of the ground state by preparing it in two different ways. One of the way to obtain the initial ground state in the left well, $V_L = \frac{1}{2}(x+2)^2 + \frac{1}{2}y^2$, of a symmetric double-well by propagating the MCTDHB equations of motion in imaginary time [1–25] with the mean-field interaction parameter $\Lambda = 0.01\pi$. In order to investigate the real-time tunneling phenomenon of the ground state, we suddenly change the trapping potential to a symmetric double-well $V_T(x, y)$ (see the main text). The wavefuntion of the ground state prepared in this way by quenching only the trapping potential is referred to as Ψ_{IG} in Fig. S10. Another way to produce the

ground state is considering a non-interacting Gaussian wavefunction in the left well of a symmetric double-well, and simultaneously quench the mean-field interaction from $\Lambda = 0$ to $\Lambda = 0.01\pi$ and trap potential from $V_L(x, y)$ to $V_T(x, y)$. The wavefunction of the ground state obtained by the later process is termed as Ψ_{NIG} in Fig. S10. The second process is applied to produce the ground, excited and vortex states in the main text. We have computed the different physical quantities at the many-body level of the ground state using two different procedures with $M = 6$ time-adaptive orbitals. The many-body Hamiltonian is represented by 64×64 exponential discrete-variable-representation grid points in a box size $[-10, 10) \times [-10, 10)$.

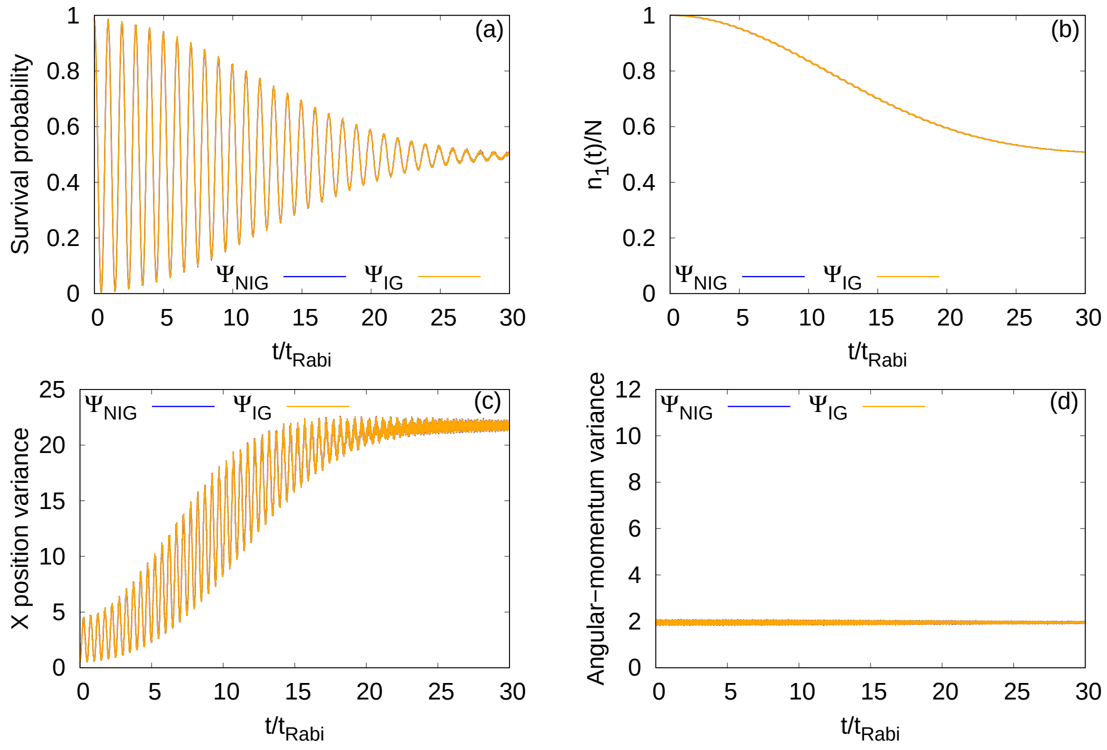

FIG. S10. Equivalence of the (a) survival probability, (b) occupation of the first natural orbital, and variances of the (c) position operator and (d) angular-momentum operator for $M = 6$ time-adaptive orbitals of the ground state, prepared in two different ways. Here Ψ_{NIG} and Ψ_{IG} refer to the non-interacting and interacting initial ground state, respectively. See the text for more details. The quantities shown are dimensionless.

In Fig. S10, we demonstrate the numerical equivalence of the long-time survival probability, $P_L(t)$, the loss of coherence in terms of occupation number of the first natural orbital, $\frac{n_1(t)}{N}$, position variance along the x-direction, $\frac{1}{N}\Delta^2_{\hat{X}}(t)$, and angular-momentum variance, $\frac{1}{N}\Delta^2_{\hat{L}_Z}(t)$, of the ground state computed using two different procedures presented above. We have already discussed in the main text that the variance of any quantum operator is much more sensitive to the many-body effects compared to the survival probabilities and the loss of coherence of the state.

Moreover, as the angular-momentum is the combination of the position and momentum, $\frac{1}{N}\Delta^2_{\hat{L}_Z}(t)$ is the most sensitive and it requires more numerical resources compared to the other quantities discussed in this work. Therefore, the convergences of $\frac{1}{N}\Delta^2_{\hat{X}}(t)$ and $\frac{1}{N}\Delta^2_{\hat{L}_Z}(t)$ automatically imply the convergences of the $P_L(t)$ and $n_1(t)$.

Fig. S10 shows the complete overlap of $P_L(t)$, $\frac{n_1(t)}{N}$, $\frac{1}{N}\Delta^2_{\hat{X}}(t)$, and $\frac{1}{N}\Delta^2_{\hat{L}_Z}(t)$ of the ground state computed using two different procedures. As there are very small oscillations occurring with time for the variances of the position and momentum operators along the y-direction for the ground state, we have not shown them here explicitly. We have verified that the respective curves sit atop each other as well. Based on the consistency of the dynamics when the ground state is prepared in the two different ways, we have further investigated the dynamics of the excited and vortex states in the main text. The long-time dynamics in Fig. S10 shows that the collapse in $P_L(t)$ is consistent with the equilibration of $\frac{n_1(t)}{N}$, $\frac{1}{N}\Delta^2_{\hat{X}}(t)$, and $\frac{1}{N}\Delta^2_{\hat{L}_Z}(t)$.

IV. COMPARISON OF TWO DIMENSIONS AND CORRESPONDING ONE DIMENSION ANALOGS OF THE GROUND AND LONGITUDINALLY-EXCITED STATES

Here, we make a comparison study of the quantities discussed in the main text for the ground and longitudinally-excited states with their one-dimensional analogs. At first, we briefly present the derivation to move from two dimensions to one dimension to decouple the transverse direction from the longitudinal direction. Then, we graphically demonstrate the comparison of the two-dimensional results with their corresponding one-dimensional analogs both at the mean-field and many-body levels.

One assumes that there are no excitations in the transverse direction. Namely, that the transverse contribution of each particle to the many-particle wave-function is given by the non-interacting ground state in the transverse direction, $\phi_0(y)$. Explicitly, the many-boson wavefunction [in Eq (2) of the main text] is given by the following ansatz

$$\Psi(\mathbf{r}_1, ..., \mathbf{r}_N; t) = \phi_0(y_1) \times ... \times \phi_0(y_N) \times \Psi_{1\mathrm{D}}(x_1, ..., x_N; t). \tag{4.1}$$

Plugging Eq. 4.1 into the two-dimensional time-dependent Schrödinger equation [Eq. (1) from

main text] and integrating over $y_1, ..., y_N$, we get

$$\hat{H}_{1\mathrm{D}}\Psi_{1\mathrm{D}} = i\frac{\partial \Psi_{1\mathrm{D}}}{\partial t}, \qquad \hat{H}_{1\mathrm{D}}(x_1, x_2, \ldots, x_N) = \sum_{j=1}^{N} \hat{h}_{1\mathrm{D}}(x_j) + \sum_{j<k} \hat{W}_{1\mathrm{D}}(x_j - x_k). \tag{4.2}$$

Here, $\hat{h}_{1\mathrm{D}}(x) = \hat{T}_{1\mathrm{D}}(x) + \hat{V}_{1\mathrm{D}}(x)$ is the one-particle Hamiltonian where $\hat{T}_{1\mathrm{D}}(x)$ and $\hat{V}_{1\mathrm{D}}(x)$ represent the kinetic energy and trap potential, respectively, and irrelevant transverse-energy term (which is a constant) is omitted. $\hat{V}_{1\mathrm{D}}(\mathbf{x})$ takes the form in one dimension as

$$V_{1\mathrm{D}}(x) = \begin{cases} \frac{1}{2}(x+2)^2, & x < -\frac{1}{2}, \\ \frac{3}{2}(1-x^2), & |x| \leq \frac{1}{2}, \\ \frac{1}{2}(x-2)^2, & x > +\frac{1}{2}, \end{cases} \tag{4.3}$$

and

$$\hat{W}_{1\mathrm{D}}(x_1 - x_2) = \int dy \int dy' |\phi_0(y)|^2 |\phi_0(y')|^2 \hat{W}(\mathbf{r}_1 - \mathbf{r}_2). \tag{4.4}$$

The dynamics of the one-dimensional many-particle Schrödinger equation, Eq. 4.2, is solved at the many-body and mean-field levels of theory, and compared to the respective dynamics in two dimensions in Figs. S11 to S15. For the Gaussian interaction, $W(\mathbf{r}_1 - \mathbf{r}_2) = \lambda_0 \frac{e^{-(\mathbf{r}_1 - \mathbf{r}_2)^2/2\sigma^2}}{2\pi\sigma^2}$, and for the transverse harmonic confinement, $\phi_0(y) = 1/(\pi)^{1/4} e^{-y^2/2}$, Eq. 4.4 reads explicitly

$$\hat{W}_{1\mathrm{D}}(x_1 - x_2) = \lambda_{0,1\mathrm{D}} \frac{e^{-(x_1 - x_2)^2/2\sigma^2}}{\sqrt{2\pi\sigma^2}} \tag{4.5}$$

with $\lambda_{0,1\mathrm{D}} = \frac{1}{\sqrt{1+\sigma^2}} \frac{\lambda_0}{\sqrt{2\pi}}$. Finally, we note that the mean-field dynamics in one dimension is computed from the Gross-Pitaevskii equation

$$\left[\hat{h}_{1\mathrm{D}} + (N-1) \int dx' |\phi_{1\mathrm{D}}(x';t)|^2 \hat{W}_{1\mathrm{D}}(x - x')\right] \phi_{1\mathrm{D}}(x;t) = i\frac{\partial \phi_{1\mathrm{D}}(x;t)}{\partial t}. \tag{4.6}$$

The latter is equivalently obtained by using the ansatz for the Gross-Pitaevskii orbital, $\phi(\mathbf{r};t) = \phi_0(y) \times \phi_{1\mathrm{D}}(x;t)$, plugging into the two-dimensional Gross-Pitaevskii equation, and integrating over y.

Now, we compare the survival probability, loss of coherence, details of the fragmentation, and the position and momentum variances for Ψ_G and Ψ_X found in the two-dimensional double-well potential and their respective one-dimensional analogs. The comparisons are made at the mean-field as well as the many-body levels. From Fig. S11, one can find that the plots of the survival

probabilities for two dimensions and one dimension double-wells fall on top of each other both at the mean-field and many-body levels due to the treatment of the weakly interacting system. Similar to the survival probability, the loss of coherence, appears due to the many-body correlations, shows a complete overlap both for Ψ_G and Ψ_X, see Fig. S12. As the survival probability and the occupancy of the first natural orbital are the most basic quantities, to shed further light on the many-particle wavefunction, we study how the fragmentation develops in the system and the correspondence of the position and momentum variances with the development of the fragmentation.

In Fig. S13, we plot the dynamical behavior of the natural occupations of the second, third, and fourth natural orbitals for Ψ_G and Ψ_X both in two dimensions and one dimension double-wells. The plot shows that the natural occupations of all the orbitals for Ψ_G found in the two-dimensional double-well fall on top of the respective occupations of the orbitals in the one-dimensional analog. In Fig. S13 (b), it is observed that although the natural occupation of the first excited natural orbital, $\frac{n_2(t)}{N}$, for Ψ_X is identical for the two dimensions and one dimension double-wells, the occupation of the second excited natural orbital, $\frac{n_3(t)}{N}$, shows a small deviation in the long-time dynamics with more occupancy achieved in one dimension double-well. But it is clear from the plot that the natural occupancy of $\frac{n_3(t)}{N}$ for Ψ_X is very small, less than 10^{-3}, even in the long-time dynamics.

In Figs. S14 and S15, we present the dynamics of the position and momentum variances, respectively, for Ψ_G and Ψ_X. The mean-field dynamics of the position and momentum variances for both the states completely overlap while computed for the two and one-dimensional double-wells. Following the discussion of the fragmentation dynamics, only the many-body position and momentum variances for Ψ_X show very small differences between the results found in two and one-dimensional double-wells, see Figs. S14(d) and S15(d), respectively.

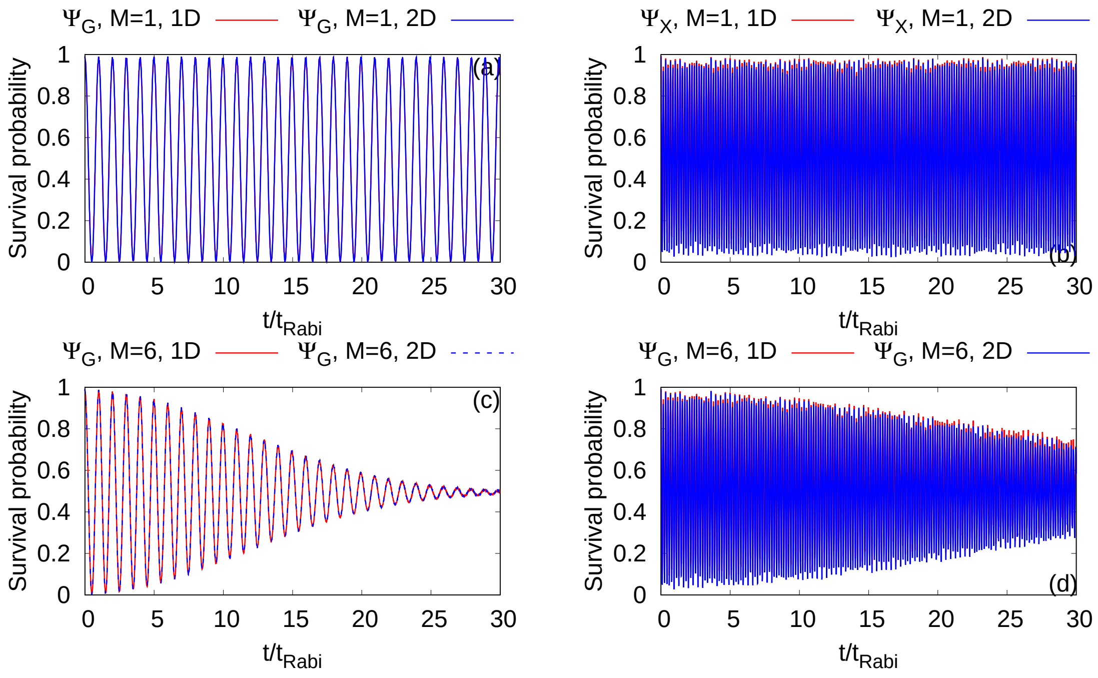

FIG. S11. Time evolution of the survival probabilities in the left well, $P_L(t)$, in a 2D symmetric double-well potential for the initial states Ψ_G and Ψ_X versus their corresponding one-dimensional analogs. Mean field results [(a) and (b)] and many-body results [(c) and (d)]. The number of bosons is $N = 10$. The many-body time evolutions are computed using the MCTDHB method with $M = 6$ time-adaptive orbitals. See the text for more details. The quantities shown are dimensionless.

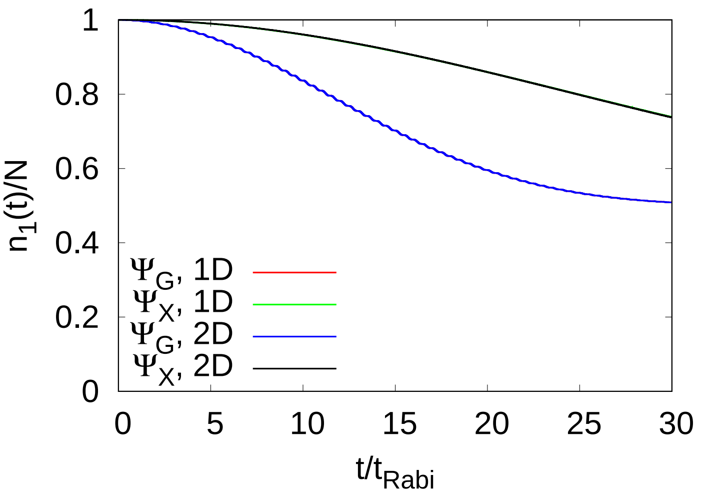

FIG. S12. Time-dependent condensate fraction, $\frac{n_1(t)}{N}$, in a symmetric 2D double-well for the initial states, Ψ_G and Ψ_X, are compared with their one-dimensional analogs. The number of bosons is $N = 10$. The many-body time evolutions are computed using the MCTDHB method with $M = 6$ time-adaptive orbitals. See the text for more details. The quantities shown are dimensionless.

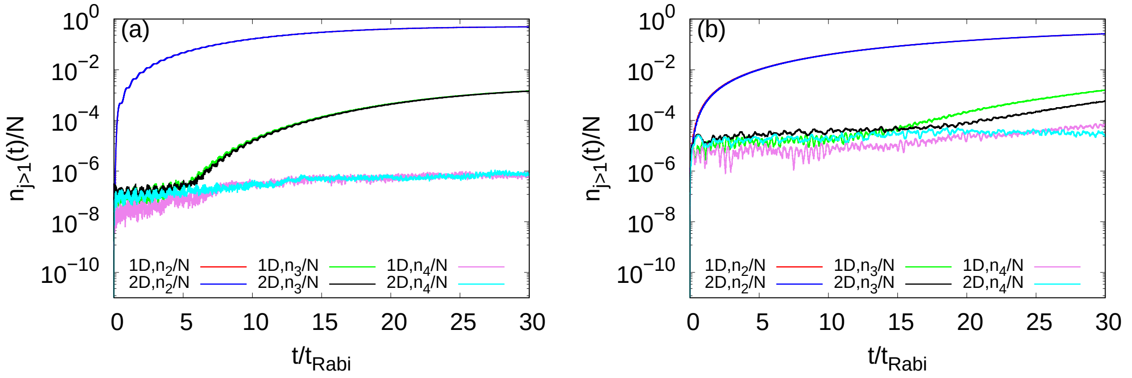

FIG. S13. Details of the depletion. Time evolution of the occupation numbers per particle of the higher natural orbitals, $\frac{n_{j>1}(t)}{N}$ with $j = 2, 3$, and 4, in a symmetric 2D double-well for the initial states (a) Ψ_G and (b) Ψ_X are compared with their one-dimensional analogs. The number of bosons is $N = 10$. The many-body time evolutions are computed using the MCTDHB method with $M = 6$ time-adaptive orbitals. Color codes are explained in the panels. See the text for more details. The quantities shown are dimensionless.

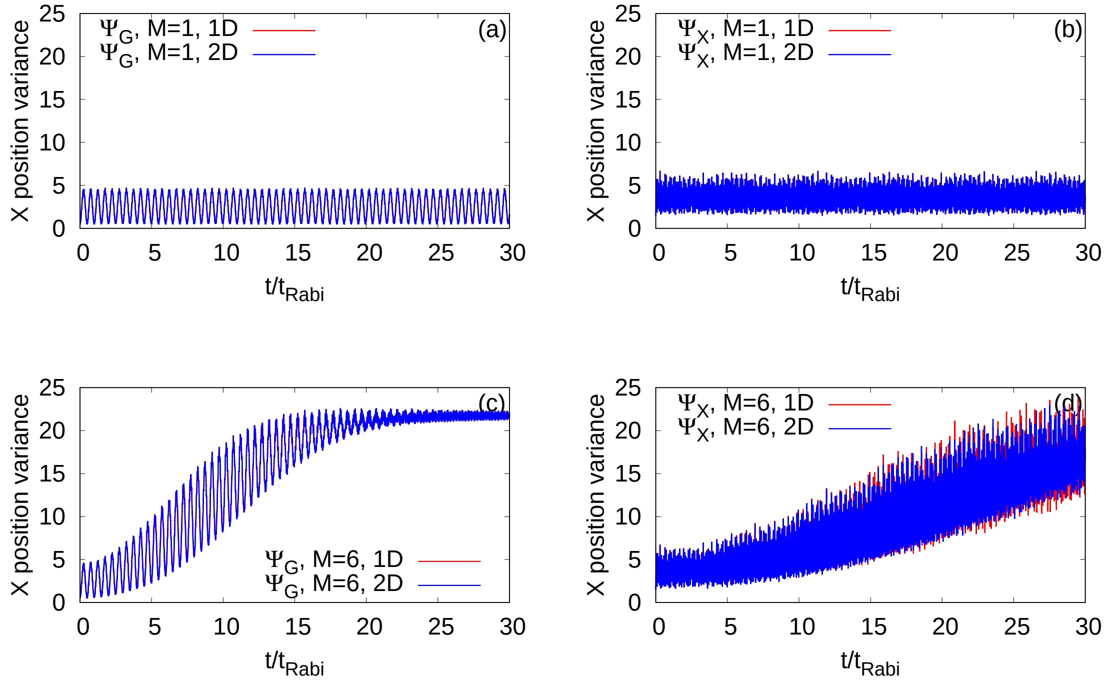

FIG. S14. Time evolutions of the position variance per particle along the x-direction, $\frac{1}{N}\Delta^2_{\hat{X}}(t)$, in a 2D symmetric double-well potential for the initial states Ψ_G and Ψ_X versus their one-dimensional analogs. Mean-field results [(a) and (b)] and the many-body results [(c) and (d)]. The number of bosons is $N = 10$. The many-body time evolutions are computed using the MCTDHB method with $M = 6$ time-adaptive orbitals. See the text for more details. The quantities shown are dimensionless.

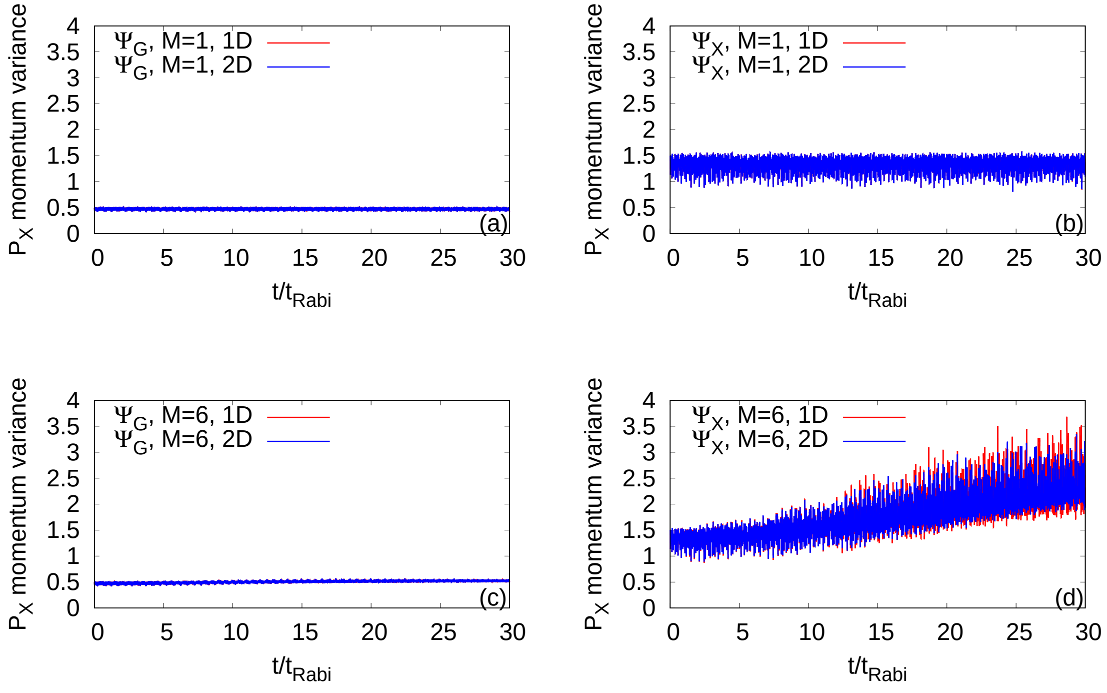

FIG. S15. Time evolutions of the momentum variance per particle along the x-direction, $\frac{1}{N}\Delta^2_{\hat{P}_X}(t)$, in a 2D symmetric double-well potential for the initial states Ψ_G and Ψ_X versus their one-dimensional analogs. Mean-field results [(a) and (b)] and the many-body results [(c) and (d)]. The number of bosons is $N = 10$. The many-body time evolutions are computed using the MCTDHB method with $M = 6$ time-adaptive orbitals. See the text for more details. The quantities shown are dimensionless.

[1] Alon, O. E. Analysis of a Trapped Bose-Einstein Condensate in Terms of Position, Momentum, and Angular-Momentum Variance. Symmetry **11**, 1344 (2019).

[2] Lode, A. U. J., Lévêque, C., Madsen, L. B., Streltsov, A. I. & Alon, O. E. Colloquium: Multiconfigurational time-dependent Hartree approaches for indistinguishable particles. Rev. Mod. Phys. **92**, 011001 (2020).

[3] Klaiman, S., Streltsov, A. I. & Alon, O. E. Uncertainty product of an out-of-equilibrium many-particle system. Phys. Rev. A **93**, 023605 (2016).

[4] Streltsov, A. I., Alon, O. E. & Cederbaum, L. S. Role of excited states in the splitting of a trapped interacting Bose-Einstein condensate by a time-dependent barrier. Phys. Rev. Lett. **99**, 030402 (2007).

[5] Alon, O. E., Streltsov, A. I. & Cederbaum, L. S. Multiconfigurational time-dependent Hartree method for bosons: many-body dynamics of bosonic systems. Phys. Rev. A **77**, 033613 (2008).

[6] Grond, J., Schmiedmayer, J. & Hohenester, U. Optimizing number squeezing when splitting a mesoscopic condensate. Phys. Rev. A **79**, 021603(R) (2009).

[7] Grond, J., Betz, T., Hohenester, U., Mauser, N. J., Schmiedmayer, J. & Schumm, T. The Shapiro effect in atom chip-based bosonic Josephson junctions. New J. Phys. **13**, 065026 (2011).

[8] Streltsov, A. I. Quantum systems of ultracold bosons with customized interparticle interactions. Phys. Rev. A **88**, 041602(R) (2013).

[9] Streltsova, O. I., Alon, O. E., Cederbaum, L. S. & Streltsov, A. I. Generic regimes of quantum many-body dynamics of trapped bosonic systems with strong repulsive interactions. Phys. Rev. A **89**, 061602(R) (2014).

[10] Klaiman, S., Lode, A. U. J., Streltsov, A. I., Cederbaum, L. S. & Alon, O. E. Breaking the resilience of a two-dimensional Bose-Einstein condensate to fragmentation. Phys. Rev. A **90**, 043620 (2014).

[11] Fischer, U. R., Lode, A. U. J. & Chatterjee, B. Condensate fragmentation as a sensitive measure of the quantum many-body behavior of bosons with long-range interactions. Phys. Rev. A **91**, 063621 (2015).

[12] Tsatsos M. C. & Lode, A. U. J. Resonances and dynamical fragmentation in a stirred Bose-Einstein condensate. J. Low Temp. Phys. **181**, 171 (2015).

[13] Schurer, J. M., Negretti, A. & Schmelcher, P. Capture dynamics of ultracold atoms in the presence of an impurity ion. New J. Phys. **17**, 083024 (2015).

[14] Lode, A. U. J. & Bruder, C. Dynamics of Hubbard Hamiltonians with the multiconfigurational time-dependent Hartree method for indistinguishable particles. Phys. Rev. A **94** 013616 (2016).

[15] Weiner, S. E., Tsatsos, M. C., Cederbaum, L. S. & Lode, A. U. J. Phantom vortices: hidden angular momentum in ultracold dilute Bose-Einstein condensates. Sci Rep **7**, 40122 (2017).

[16] Lode, A. U. J. & Bruder, C. Fragmented superradiance of a Bose-Einstein condensate in an optical cavity. Phys. Rev. Lett. **118**, 013603 (2017).

[17] Lode, A. U. J., Diorico. F. S., Wu, R., Molignini, P., Papariello, L., Lin, R., Lévêque, C., Exl, L., Tsat-

sos, M. C., Chitra, R. & Mauser, N. J. Many-body physics in two-component Bose-Einstein condensates in a cavity: fragmented superradiance and polarization. New J. Phys. **20**, 055006 (2018).

[18] Klaiman, S., Beinke, R., Cederbaum, L. S., Streltsov, A. I. & Alon, O. E. Variance of an anisotropic Bose-Einstein condensate. Chemical Physics **509**, 45 (2018).

[19] Alon, O. E. & Cederbaum, L. S. Attractive Bose-Einstein condensates in anharmonic traps: Accurate numerical treatment and the intriguing physics of the variance. Chemical Physics **515**, 287 (2018).

[20] Chatterjee, B., Tsatsos, M. C. & Lode, A. U. J. Correlations of strongly interacting one-dimensional ultracold dipolar few-boson systems in optical lattices. New J. Phys. **21**, 033030 (2019).

[21] Alon, O. E. Condensates in annuli: dimensionality of the variance. Molecular Physics **117**, 2108 (2019).

[22] Bera, S., Chakrabarti, B., Gammal, A., Tsatsos, M. C., Lekala, M. L., Chatterjee, B., Lévêque, C. & Lode, A. U. J. Sorting Fermionization from Crystallization in Many-Boson Wavefunctions. Sci. Rep. **9**, 17873 (2019).

[23] Lin, R., Molignini, P., Papariello, L., Tsatsos, M. C., Lévêque, C., Weiner, S. E., Fasshauer, E., Chitra, R. & Lode A. U. J. MCTDH-X: The multiconfigurational time-dependent Hartree method for indistinguishable particles software. Quantum Sci. Technol. **5**, 024004 (2020).

[24] Streltsov, A. I. & Streltsova, O. I. 2015 MCTDHB-Lab, version 1.5, 2015 (http://mctdhb-lab.com).

[25] Streltsov, A. I., Cederbaum, L. S., Alon, O. E., Sakmann, K., Lode, A. U. J., Grond, J., Streltsova, O. I., Klaiman, S. & Beinke, R. The Multiconfigurational Time-Dependent Hartree for Bosons Package, Version 3.x, http://mctdhb.org.
